# Automated Annotation of Sites of Metabolism from Biotransformation Data

**DOI:** 10.1021/acs.jcim.5c00819

**Published:** 2025-06-17

**Authors:** Roxane Axel Jacob, Angelica Mazzolari, Johannes Kirchmair

**Affiliations:** † Department of Pharmaceutical Sciences, Division of Pharmaceutical Chemistry, Faculty of Life Sciences, 27258 University of Vienna, Josef-Holaubek-Platz 2, 1090 Vienna, Austria; ‡ Christian Doppler Laboratory for Molecular Informatics in the Biosciences, Department of Pharmaceutical Sciences, 27258 University of Vienna, Josef-Holaubek-Platz 2, 1090 Vienna, Austria; ¶ Dipartimento di Scienze Farmaceutiche, 60232 Universita degli Studi di Milano, I-20133 Milano, Italy; § Christian Doppler Laboratory for Molecular Informatics in the Biosciences, Department of Pharmaceutical Sciences, 27258 University of Vienna, Josef-Holaubek-Platz 2, 1090 Vienna, Austria; ∥ Vienna Doctoral School of Pharmaceutical, Nutritional and Sport Sciences, 27258 University of Vienna, Josef-Holaubek-Platz 2, 1090 Vienna, Austria

## Abstract

Computational models predicting the Sites-of-Metabolism (SOMs) of small organic molecules have become invaluable tools for studying and optimizing the metabolic properties of xenobiotics. However, the performance of SOM predictors has shown signs of plateauing in recent years, primarily due to the limited availability of training data. While vast amounts of biotransformation data in the form of substrate–metabolite pairs exist, their potential for SOM prediction remains largely untapped due to the absence of annotations. Annotating SOMs requires expert knowledge and is a highly time-consuming process. To address this challenge, we introduce AutoSOM, the first open-source tool that automatically extracts SOMs by mapping structural differences using transformation rules. AutoSOM is both fast and highly accurate, achieving over 90% labeling accuracy on a diverse validation set of more than 5,000 reactions within minutes. Moreover, its annotation process is fully transparent and interpretable, which we hope will facilitate its adoption in high-stakes downstream applications such as drug discovery campaigns and regulatory assessments. Beyond accelerating annotation, AutoSOM enables standardized and consistent SOM labeling across institutions without requiring direct data sharing.

## Graphical abstract

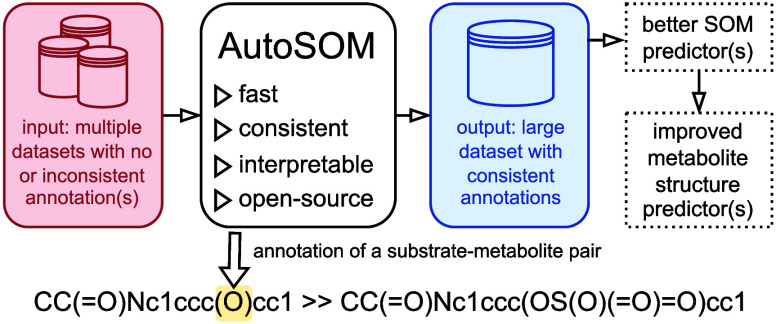

## 1 Introduction

Computational tools for metabolism prediction have emerged as valuable aids for assessing the metabolic fate of xenobiotics, enabling faster and more cost-effective evaluations.^1–7^ Among them, SOM predictors identify the specific atoms where metabolic reactions occur and are used as standalone models^8–15^ or in conjunction with biotransformation rules to predict likely metabolites.^16–19^ However, despite ongoing efforts to improve SOM prediction, progress has plateaued, likely due to the limited quantity and quality of available training data. This shortage is reflected in the fact that most of the available SOM predictors rely on just two data sets, encompassing approximately 680 ^20^ and 2000 ^21^ substrates, respectively.

Fortunately, vast amounts of potential training data exist in the form of unannotated substrate-metabolite pairs. However, because manual SOM annotation is labor-intensive and requires expert evaluation,^21^ they remain untapped. Automating the SOM annotation process would dramatically increase the amount of available training data, thus improving SOM predictors and, by extension, metabolite structure predictors.^17,19,22^

To this end, previous studies have predominantly used Maximum Common Substructure (MCS) matching to generate Atom-to-Atom-Mappings (AAMs) between substrates and metabolites, enabling the identification of reaction centers by detecting atoms with altered connectivity.^10,23,24^ While effective for simple transformations involving atom additions, such as oxidation, hydroxylation, and conjugation, this approach fails to produce chemically meaningful SOM annotations for reactions involving atom removal, such as dealkylation. Finkelmann et al.^11^ sought to address this limitation by incorporating reaction type information, but their method relies on prior reaction classification, which is often unavailable. Additionally, MCS matching struggles with complex rearrangements, ring-breaking, or ring-closing reactions. More advanced AAM methods^25^ present a potential alternative but often underperform in metabolic reaction data due to the omission of key reactants from reaction records.

In this work, we introduce AutoSOM, the first open-source software for extracting chemically meaningful SOMs from substrate-metabolite pairs without requiring prior knowledge of reaction types (Figure 1). AutoSOM identifies SOMs by mapping structural differences between substrate and metabolite molecules to a set of general annotation rules. We rigorously evaluate AutoSOM’s labeling accuracy across two distinct data sets and demonstrate its potential for mobilizing previously unused metabolism data and identifying inconsistencies and errors in existing metabolism databases.

**Figure 1:**
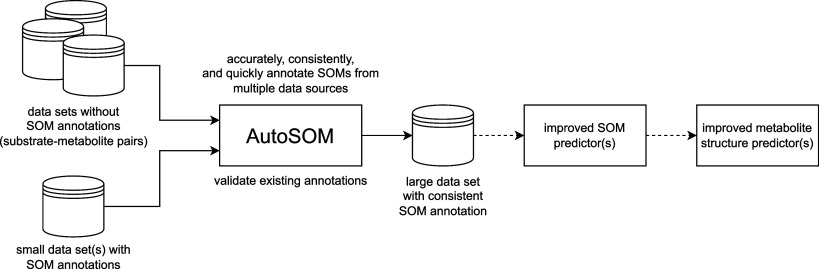
AutoSOM efficiently generates accurate and consistent SOM annotations from substrate-metabolite pairs and can be used to support the manual validation of existing annotations. The annotated data can then be used to train SOM prediction models, ultimately improving metabolite structure prediction.

## 2 Related Work

### 2.1 Annotating Reaction Centers of General Organic Chemical Reactions

Chemical reaction centers, also known as reacting centers, are defined as either the bonds that are broken or formed during a reaction, or as the reactant atoms involved in bond and electron rearrangements.^26^ Identifying these centers requires computing an AAM between reactants and products. Algorithms for generating these AAMs typically fall into the following four categories:

#### 2.1.1 Fragment-Based Methods

These approaches break reacting molecules into fragments, discard unchanged fragments, and reconstruct reaction centers from the remaining atoms.^27,28^

#### 2.1.2 Common Substructure-Based Methods

This category includes methods that identify the maximum common subgraph^29,30^ or the maximum common edge subgraph^31^ between reactants and products. These methods are further classified into two subcategories. In the first, reaction centers are defined as the bonds that are broken or formed within the MCS^29^ or as the atoms directly involved in those bonds.^30^ In the second subcategory, reaction centers are determined by detecting atoms with altered structural fingerprints, typically computed using the Morgan algorithm^32^ or its variants.^30,33,34^

#### 2.1.3 Optimization-Based Methods

This category includes approaches relying on the principle of minimal chemical distance, which assumes that chemical reactions involve breaking and forming the fewest possible bonds. These methods either directly solve the graph isomorphism problem,^35,36^ use the A* graph-traversal and pathfinding algorithm,^37^ or employ integer linear optimization methods^38,39^ to find the reaction centers.

For a comprehensive review of the first three categories of AAM algorithms, we refer the reader to the work of Chen et al.^26^ Problems with these methods include the fact that they struggle with complex reactions, such as 1,2-rearrangements, and often fail in cases where multiple algorithmically valid solutions exist but only one is chemically correct.^40^ To address this shortcoming, Jaworski et al.^40^ proposed introducing expert-curated reaction templates to generate plausible intermediate atom assignments that are used to guide a graph-theoretical algorithm toward chemically correct isomorphic mappings.

#### 2.1.4 Machine Learning-Based Methods

The fourth category employs various machine-learning algorithms to generate AAMs. The first prominent tool to achieve excellent mapping accuracy on a benchmark set of synthetic organic reactions^41^ was RXNMapper,^25^ a transformer neural network model. Recently, LocalMapper,^42^ a Graph Neural Network (GNN)-based model that learns AAMs from chemist-labeled reactions via human-in-the-loop machine learning, has been reported to outperform RXNMapper on a manually curated subset of the USPTO-50k data set.^41^ Enabled by the availability of large reaction databases with AAM annotations for training, these approaches achieve state-of-the-art performance in mapping the atoms of stoichiometrically balanced chemical reactions.

### 2.2 Annotating Sites of Metabolism

SOMs can be considered the equivalent of reaction centers in enzyme-catalyzed metabolic reactions. In the literature, SOMs are typically defined in one of two ways: (1) as the substrate atoms involved in bond and electron rearrangements, i.e., the atoms undergoing topological changes in a molecular graph^10,11,15,23,24^ or (2) as the atoms where metabolic reactions are initiated.^13,14,20,21,43^ The latter definition generates chemically meaningful SOM labels that align with the principles of chemical reactivity and enzyme catalysis. Expert-annotated data sets^20,21^ generally follow the latter definition. An example illustrating these different annotation strategies is shown in Figure 2.

**Figure 2:**
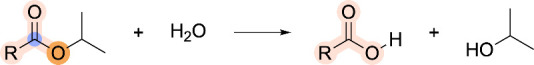
Example of the discrepancy between automated, MCS-based SOM annotations and expert-derived, chemically meaningful SOM annotations. The MCS between the substrate and metabolite is highlighted in beige. Comparing the molecular graphs of the substrate and metabolite, we see that the atom experiencing connectivity changes during enzymatic ester hydrolysis is the *sp* ^3^-hybridized oxygen, highlighted in orange. MCS-based annotation strategies identify this oxygen atom as the SOM. However, ester hydrolysis reactions are initiated at the carbonyl carbon, highlighted by a blue circle, which chemically meaningful, expert-derived annotation strategies designate as the SOM.

To the best of our knowledge, no dedicated tool exists for extracting chemically meaningful SOM annotations from substrate-metabolite pairs. However, some automated SOM annotation strategies have already been implemented, mainly as preprocessing steps for generating data for training SOM prediction models.^10,11,15,23,24^ These have uniformly followed the first definition (i.e., detecting topological differences in molecular graphs) due to its simplicity.

Several studies have used variations of MCS mapping to extract SOMs from the discontinued BIOVIA Metabolite Database. Boyer et al.^23^ identified SOMs by generating rooted circular atom fingerprints, computing the MCS between substrates and metabolites, and detecting atoms with altered fingerprints. Similarly, Rudik et al.^10^ applied MCS mapping to annotate reaction centers in cytochrome P450 (CYP) and uridine 5′-diphospho-glucuronosyltransferase (UGT)-mediated reactions from the BIOVIA Metabolite Database, identifying atoms whose local connectivity had changed. Adams^24^ refined the MCS mapping by incorporating heuristics to preserve ring membership, thus improving the accuracy of MCS-based AAM for metabolic reactions. While differing in implementation details, all three methods rely on MCS-based comparison to detect reaction centers. MCS-based comparison works well for most oxidation, hydroxylation, and conjugation reactions. However, the approach fails to produce chemically meaningful annotations for more complex reactions, such as dealkylation reactions or carnitine conjugation.

Finkelmann et al.^11^ addressed the challenge of distinguishing SOMs in reactions that involve either addition to or removal from the substrate. They also used data from the BIOVIA Metabolite Database and followed a protocol that involved: (1) applying AAM mapping between reactants and products (method unspecified), (2) identifying the reactive bond (i.e., the bond that was cleaved, added, or altered), (3) mapping its adjacent atoms, and (4) defining one or both adjacent atoms as SOMs based on the reaction type. For example, in alkene-to-alkane reductions, they labeled both atoms of the reactive bond as SOMs. In contrast, in oxidation or hydroxylation reactions, they designated the nonoxygen atom adjacent to the reactive bond as the SOM. Unfortunately, this approach requires prior knowledge of the reaction type, which is often unavailable in metabolic data sets.

Finally, Porokhin et al.^15^ sourced SOM annotations from the KEGG database. In their approach, reaction centers are premapped by the database’s curators.

All the aforementioned SOM annotation tools rely, to some extent, on MCS mapping, which has limitations. An alternative approach would be to use atom-mapping strategies beyond MCS, such as machine learning-based methods, which currently represent the state-of-the-art for atom mapping in organic reactions. However, our findings indicate that machine-learning-based AAM tools occasionally yield results that are inferior to those derived from expert annotations (see Figure 3). One reason for this discrepancy is that metabolic reactions are typically recorded as stoichiometrically imbalanced equations. For instance, in aliphatic hydroxylation reactions, only the substrate and hydroxylated metabolite are typically documented, while the reactant (water) is omitted. This omission confounds traditional atom-mapping tools, leading to incorrect atom assignments. As a result, metabolite atoms may be erroneously mapped to the wrong substrate atoms, resulting in inaccurate SOM annotations.

**Figure 3:**
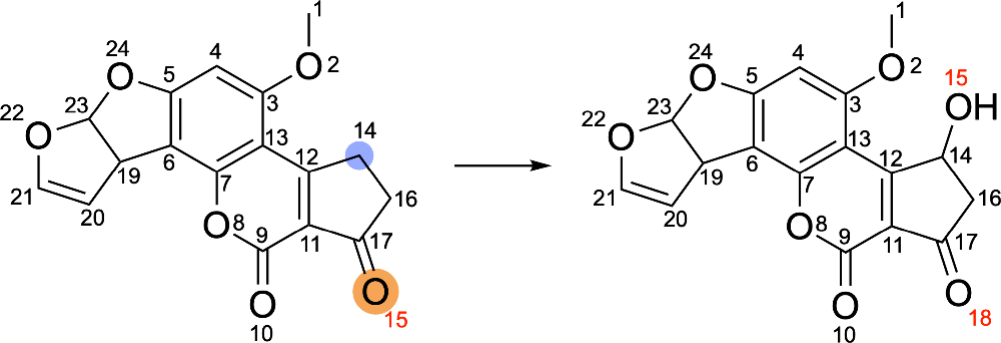
Example of incorrect AAM generated by RXNMapper. In the hydroxylation of aflatoxin B1, the oxygen atom labeled as 15 in the substrate is incorrectly mapped to the newly introduced hydroxyl oxygen in the metabolite. As a result, oxygen 15 is mistakenly identified as a SOM, as it appears to change from being doubly bonded to a carbon to being singly bonded. Incorrectly mapped atoms are highlighted by red atom numbers, the correct SOM is indicated by a blue circle, and the erroneously assigned SOM is highlighted by an orange circle.

A concrete example of this issue is the atom-mapping of aflatoxin B1 to its hydroxylated metabolite by RXNMapper,^25^ a transformer-based AAM tool (Figure 3). Trained primarily on stoichiometrically balanced organic reactions, RXNMapper struggles when key reactants, such as water in this case, are missing from the reaction record. Consequently, the oxygen atom 15 in the substrate is incorrectly mapped to the newly introduced hydroxyl oxygen in the metabolite. As a result, oxygen 15 will mistakenly be labeled as a SOM, since it appears as though it changed from being bonded to a carbon atom via a double bound to being bond to a carbon atom by a single bond.

Instead of relying solely on MCS mapping or machine-learning-based AAM to label all atoms undergoing topological changes as SOMs, AutoSOM aims to generate chemically meaningful SOM labels that better reflect the principles of chemical reactivity and enzyme catalysis without prior knowledge of the reaction type.

## 3 Methods

AutoSOM takes in substrate-metabolite pairs and annotates the SOMs of the reaction. It is implemented in Python and relies mainly on the RDKit^44^ and NetworkX^45^ packages. AutoSOM starts by preprocessing the data and performing validity checks, details of which are presented in the next paragraph. The processed data is then annotated according to the pipeline described in this section.

### 3.1 Input and Preprocessing Pipeline

AutoSOM processes substrate–metabolite pairs provided in a Comma-Separated Value (CSV) file, where each row must include the SMILES representations and unique numerical identifiers of the substrate and its corresponding metabolite. Importantly, metabolic reactions do not need to be stoichiometrically balanced to be processed by AutoSOM. The pipeline begins by checking the validity of the RDKit molecule objects generated from the input SMILES strings. This validation involves using RDKit’s *MolFromSmiles* and *MolToInchiKey* functions to compute the International Chemical Identifier (InChI) keys for both the substrate and the metabolite. If either molecule cannot be parsed into a valid RDKit Mol object, or if an InChI key cannot be generated, or if the substrate and metabolite share the same InChI key, the reaction is deemed invalid and is then excluded from further processing, with an appropriate message logged.

Reactions involving molecules that contain elements other than H, C, N, O, S, P, F, Cl, Br, I, B, Si, and Se are also filtered out. This step prevents the pipeline from attempting to annotate substrates containing uncommon atom types that have not been validated.

Next, the ChEMBL Structure Pipeline^46^ is employed to preprocess the molecules. Specifically, the *get_parent_mol* function from the *standardizer* module is used to remove counterions and solvents. The *standardize_mol* function is then applied to both the substrate and the metabolite to perform a series of standardization steps, including the removal of explicit hydrogen atoms, kekulization, normalization, and neutralization.

Canonical tautomers are then computed using RDKit’s *CanonicalTautomer* function from the *rdMolStandardize* module. Since this module underlies much of the functionality of the ChEMBL Structure Pipeline, the compatibility between these tools was a key reason for their selection, alongside their widespread adoption within the cheminformatics community.

### 3.2 Automated Annotation

The annotation rules are inspired by the SOM annotations observed in the MetaQSAR database, a high-quality resource containing over 6000 expert-annotated drug-like substrate-metabolite pairs spanning 101 reaction types. The reaction types are grouped into 21 reaction classes and three reaction main classes. A list of reaction classes, along with their relative frequencies in the MetaQSAR database, can be found in the Supporting Information, Section S2.

#### 3.2.1 General Workflow

The AutoSOM workflow for annotating SOMs from substrate-metabolite pairs is illustrated in Figure 4. It begins by computing the ratio of heavy atoms between the substrate and the metabolite. Based on this ratio, the reaction is classified as a potential addition reaction (if the metabolite contains more heavy atoms than the substrate) or a potential elimination reaction (if the metabolite contains fewer heavy atoms than the substrate). Here, “addition” and “elimination” specifically refer to the gain or loss of heavy atoms from the perspective of the substrate, rather than mechanistic addition or elimination reactions.

**Figure 4:**
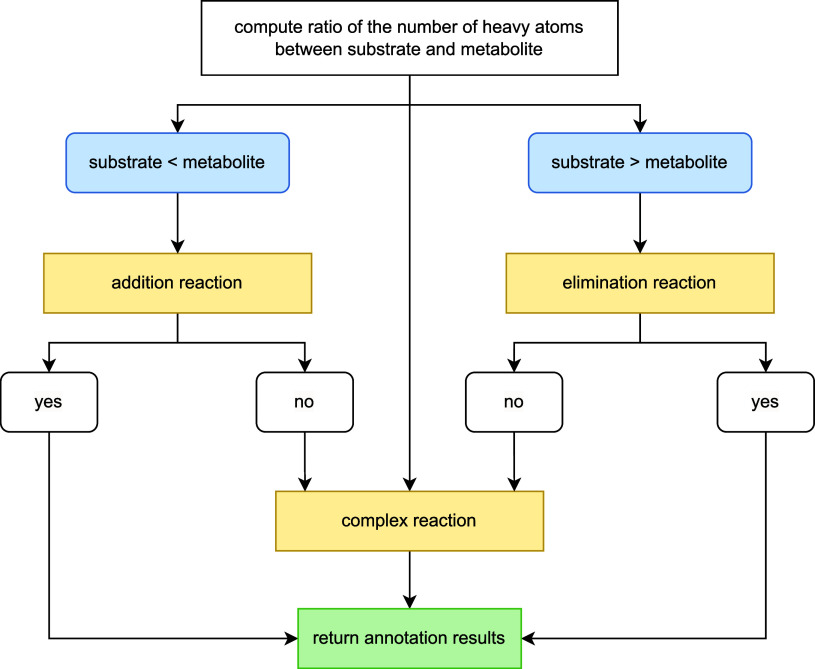
General computational workflow of AutoSOM for annotating SOMs from substrate-metabolite pairs. The pipeline begins by calculating the ratio of heavy atoms between the substrate and metabolite. Based on this ratio, reactions are classified as either potential addition or elimination reactions and processed by their respective handlers. If SOMs are identified, AutoSOM terminates and returns the annotation results. If no SOM is found, reactions are forwarded to the complex reaction handler, which annotates most remaining reactions. If no SOM is found, the reaction is returned without annotations and classified as an unknown reaction type.

Potential addition and elimination reactions are processed directly by their respective handlers. If SOMs are identified, AutoSOM terminates and returns the annotation results. If no SOM is found, reactions are forwarded to the complex reaction handler.

Finally, the complex reaction handler annotates all remaining reactions that could not be categorized in previous steps. If no SOM is identified by the complex handler, the reaction is returned without any annotations and is classified as an unknown reaction type.

To prevent performance issues caused by overly complex reactions, AutoSOM employs a timeout parameter that terminates any annotation attempt after a predefined time limit. By default, this timeout is set to 20 s. The timeout can be adjusted to suit the user’s requirements. Further details on each reaction handler are provided in the following subsections.

#### 3.2.2 Addition Reactions

The annotation of potential addition reactions is managed by the addition reaction handler. A summary of AutoSOM’s computational workflow for handling addition reactions is presented in Figure 5. An addition reaction is defined as a transformation in which the metabolite contains more heavy atoms than the substrate, and the molecular graph of the substrate is entirely contained within that of the metabolite. Examples of addition reactions include aliphatic and aromatic hydroxylations, epoxidation, oxidation, sulfation, and conjugation reactions. The first condition is verified in earlier stages of the pipeline. To confirm the second condition, the addition reaction handler checks whether the substrate is an isomorphic subgraph of the metabolite.

**Figure 5:**
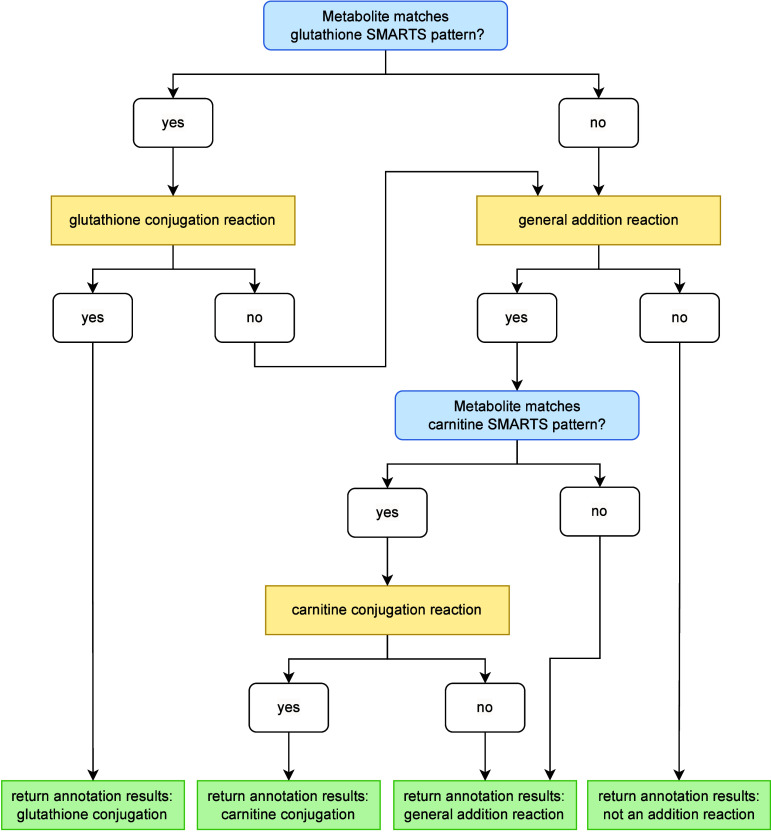
AutoSOM’s computational workflow for handling addition reactions. The process begins by checking for potential glutathione conjugation via SMARTS pattern matching. If a match is found, the glutathione handler computes and returns the SOMs. Otherwise, the reaction proceeds to the general addition reaction handler. If no SOMs are identified, it is forwarded to the complex reaction handler. If SOMs are found, the workflow concludes by verifying and applying any necessary corrections for carnitine conjugation before returning the final annotation results.

For general addition reactions, AutoSOM first applies MCS mapping to establish an AAM between the substrate and metabolite. Atoms present in both the substrate and metabolite, according to the AAM, but that acquire a new neighbor during the reaction are identified as SOMs. An example is shown in Figure 6, panel A.

**Figure 6:**
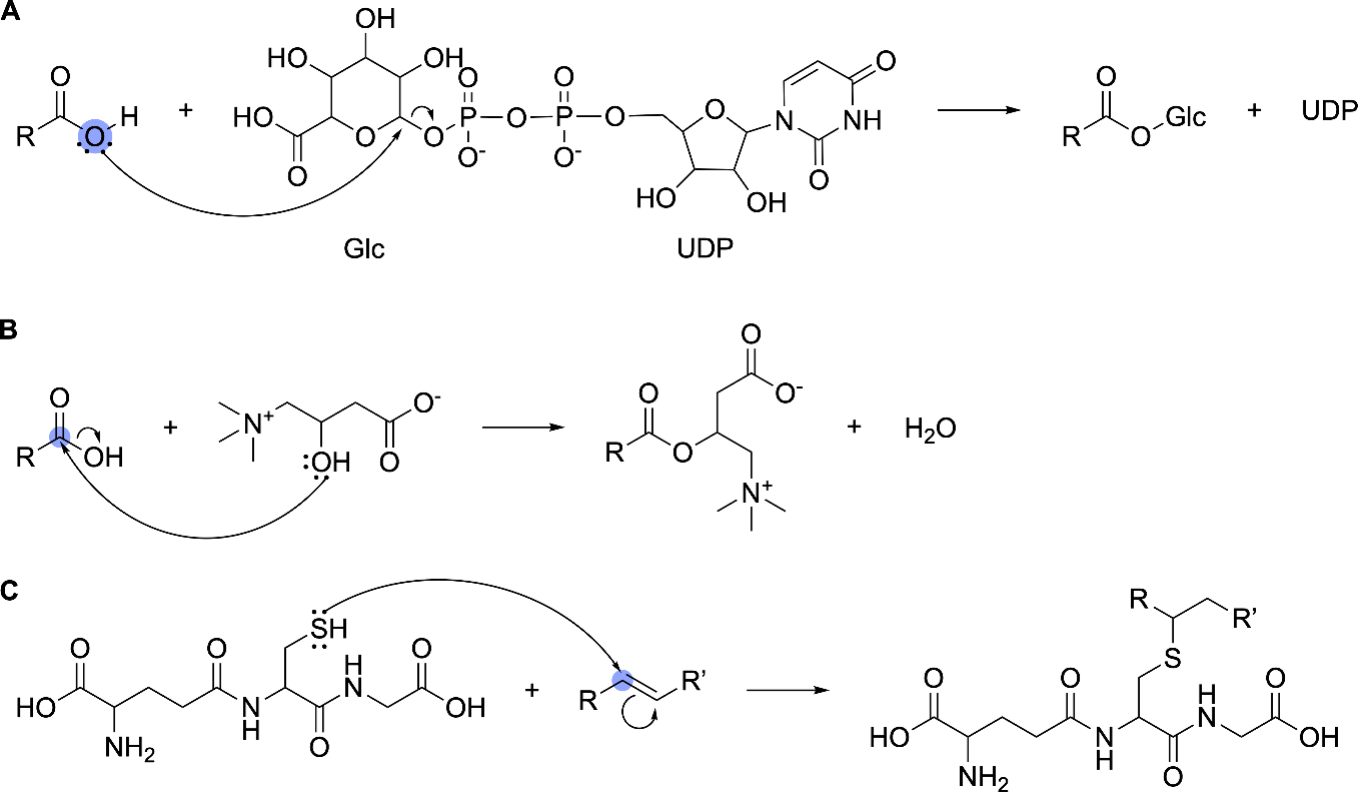
Examples of addition reaction handling. SOMs are denoted by blue circles. (A) Glucuronidation of a carboxylic acid, demonstrating how AutoSOM processes general addition reactions. (B) Carnitine conjugation, where the electrophile is part of the substrate, and the nucleophile originates from the reactant, unlike typical addition reactions. (C) Glutathione conjugation, in which both *sp* ^2^-hybridized carbon atoms undergo topological changes, but only the carbon bonded to the glutathione sulfur is designated as a SOM.

If a general addition SOM is detected, AutoSOM further evaluates whether the reaction involves the conjugation of a carnitine moiety to a carboxylic acid using SMARTS pattern matching. If so, the SOM is adjusted to indicate the carbon atom of the carboxyl group rather than the *sp* ^3^-hybridized oxygen. An example is depicted in Figure 6, panel B. This adjustment is necessary because, in carnitine conjugation, the hydroxyl group of carnitine acts as the nucleophile attacking the carboxylic acid. This contrasts with other carboxylic acid conjugations, where the *sp* ^3^-hybridized oxygen typically performs the nucleophilic attack. If an addition reaction does not match the carnitine pattern, it remains classified as a general addition.

AutoSOM processes glutathione conjugations separately due to the distinct reaction mechanisms involved. These reactions, catalyzed by glutathione S-transferase, involve the attachment of glutathione to electrophilic functional groups, such as unsaturated carbon–carbon bonds, quinones, epoxides, and aryl halides. To ensure high performance with minimal computational cost for general addition reactions, AutoSOM performs MCS matching while considering bond order (e.g., single, double, triple bonds). However, this approach is problematic for glutathione conjugations, as the electrophilic functional group to which glutathione attaches often contains a double bond, which becomes a single bond after conjugation. This hinders efficient MCS matching. To address this, AutoSOM processes glutathione conjugations separately. It first detects the presence of a glutathione moiety using SMARTS pattern matching. If identified, the correct SOM is determined through a series of isomorphic subgraph matching steps. An example is shown in Figure 6, panel C.

Reactions that do not match the patterns of either glutathione conjugation or general addition are classified as complex and forwarded to the appropriate handler for further analysis.

#### 3.2.3 Elimination Reactions

The annotation of elimination reactions in AutoSOM is handled by the elimination reaction handler. A summary of AutoSOM’s computational workflow for handling elimination reactions is presented in Figure 7. An elimination reaction is defined as a transformation in which the metabolite contains fewer heavy atoms than the substrate, and the molecular graph of the metabolite is entirely contained within that of the substrate. The first condition is verified in earlier stages of the pipeline. To verify the second condition, the elimination reaction handler checks whether the metabolite is an isomorphic subgraph of the substrate.

**Figure 7:**
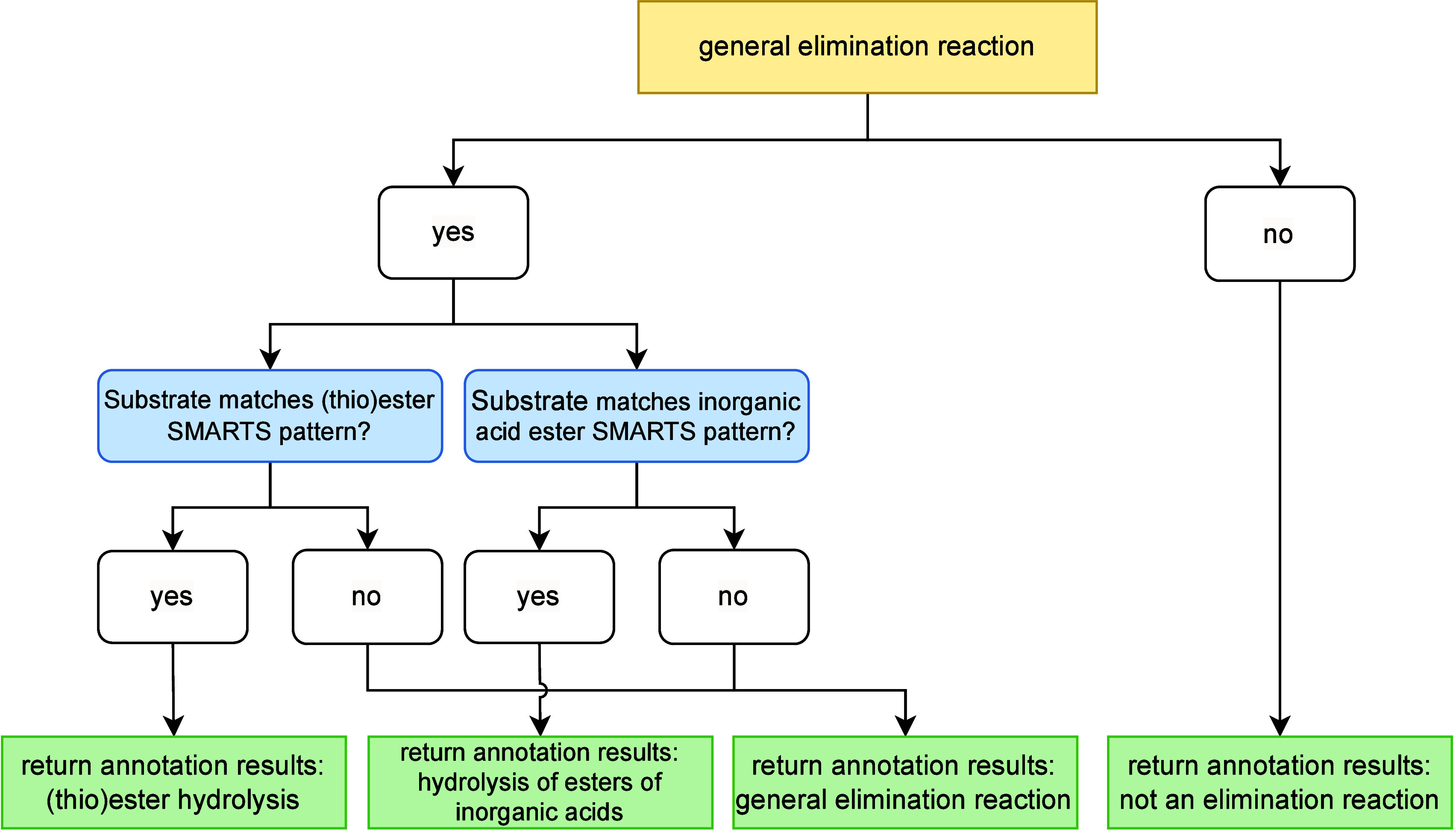
AutoSOM’s computational workflow for handling elimination reactions. The process begins by identifying SOMs for general elimination reactions. If no SOMs are found, the reaction is forwarded to the complex reaction handler. If SOMs are identified, the workflow concludes by applying any necessary corrections for the hydrolysis of (thio)­esters and esters of inorganic acids, before returning the final annotation results.

If both criteria are met, AutoSOM attempts to classify the reaction as a general elimination. To do so, it generates an AAM between the substrate and metabolite using MCS mapping. It then searches for SOMs based on two distinct patterns:

- **Type 1 general elimination reactions.** These include, for instance, dealkylation and deacylation reactions. In these cases, the SOM is an unmapped carbon atom (outside the MCS) bonded to a mapped atom (within the MCS).
- **Type 2 general elimination reactions.** These include, for example, reductive dehalogenation, reduction at heteroatoms (such as nitro or sulfoxide groups), and the reduction of alcohols to alkanes. In these cases, the SOM is the mapped atom (within the MCS) that is adjacent to an unmapped atom (outside the MCS).

Examples of both general elimination reaction types can be found in Figure 8, panels A and B. In addition to general elimination reactions, AutoSOM applies specialized handling for specific elimination cases, including:

- **(Thio)­ester hydrolysis.** The SOM is assigned to the carbonyl carbon.
- **Hydrolysis of esters of inorganic acids** (e.g., phosphates, thiophosphates, sulfamates, sulfonamides, sulfonates). The SOM is assigned to the phosphorus or sulfur atom.

**Figure 8:**
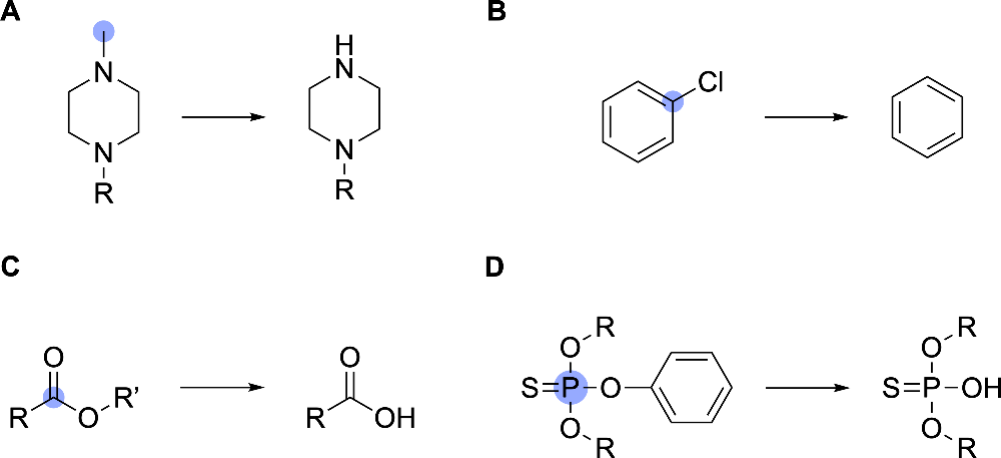
Examples of elimination reaction handling. SOMs are denoted by blue circles. (A) Demethylation of an *N*-methylpiperazine functional group, demonstrating how AutoSOM processes type 1 elimination reactions. (B) Reductive dehalogenation of chlorobenzene, illustrating type 2 elimination processing. (C) Hydrolysis of an ester functional group. (D) Hydrolysis of a thiophosphate ester.

To identify these specific elimination reactions, AutoSOM utilizes a combination of SMARTS pattern matching, MCS mapping, and molecular graph analysis. Examples can be seen in Figure 8, panels C and D. Reactions that do not match the general elimination pattern are classified as complex and forwarded to the appropriate category for further analysis.

#### 3.2.4 Complex Reactions

The annotation of complex reactions in AutoSOM is handled by the complex reaction handler. A summary of AutoSOM’s computational workflow for handling complex reactions can be found in Figure 9. This handler is designed to identify and annotate SOMs for chemical transformations that cannot be classified as simple addition or elimination reactions. These reactions are sometimes relatively simple redox reactions, but can also involve significant structural reorganization, requiring more sophisticated computational methods to determine the correct SOM. To achieve this, AutoSOM employs two main approaches for SOM identification:

**Figure 9:**
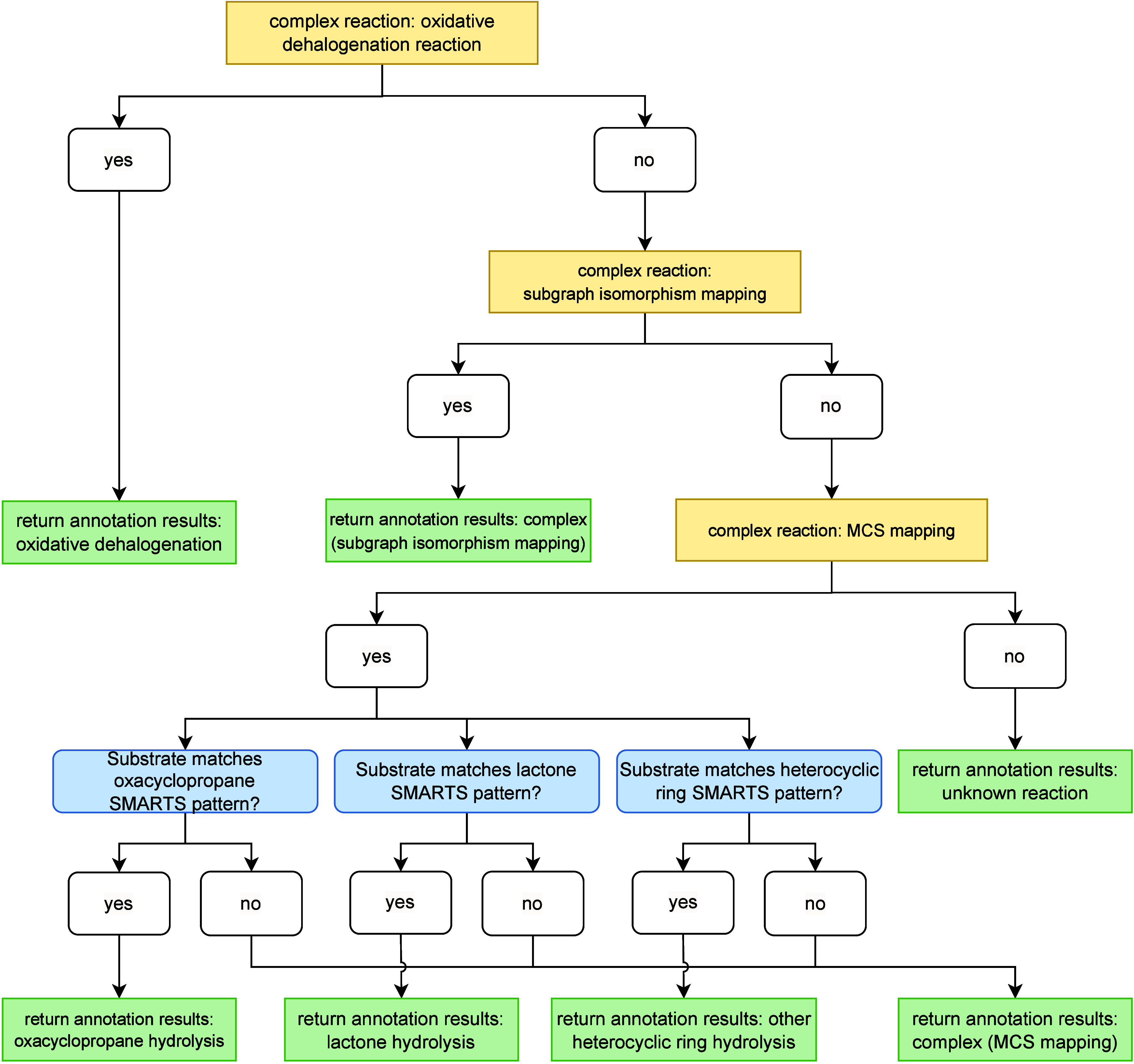
AutoSOM’s computational workflow for handling complex reactions. The process begins by identifying SOMs for potential oxidative dehalogenation. If found, the handler returns the annotation results. Otherwise, it attempts to generate a complete AAM via subgraph isomorphism. If this fails, it proceeds with MCS mapping, assigning SOMs to all atoms undergoing topological changes. If no SOMs are identified, the pipeline terminates, returning an empty SOM list and the “unknown” reaction type. If SOMs are found, the workflow concludes by applying necessary corrections for oxacyclopropane hydrolysis, lactone hydrolysis, and other heterocyclic ring hydrolyses before returning the final annotation results.

- **Subgraph isomorphism mapping.** Used when a complete mapping between the substrate and metabolite is possible. This category includes, for instance, hydrogenation or dehydrogenation reactions. In this approach, SOMs are identified by detecting atoms where the number of bonded hydrogen atoms has changed or the formal charge has changed.
- **MCS mapping.** Applied when a complete mapping cannot be established. This method identifies the largest shared substructure between the substrate and metabolite, then compares the atomic environments (neighboring atoms) of mapped atoms to pinpoint the SOMs. Every atom that underwent topological changes is labeled as a SOM.

Beyond these general methodologies, AutoSOM includes specialized handling for specific types of complex reactions:

- **Oxacyclopropane hydrolysis reactions.** In cases of a hydrolytic opening of three-membered oxygen-containing rings, the two carbon atoms in the ring are labeled as SOMs.
- **Lactone hydrolysis reactions.** In cases of a hydrolytic opening of cyclic esters, the carbonyl carbon is assigned as the SOM.
- **Other heterocyclic ring opening reactions.**

Examples for all reaction types can be found in Figure 10, panels A–E. Additionally, AutoSOM’s complex handler separately processes oxidative dehalogenation reactions, which are characterized by the retention of the same number of carbon atoms in the substrate and metabolite, the loss of a halogen atom, and the gain of an oxygen atom in the metabolite. Two distinct types of oxidative dehalogenation metabolites are considered. In the first type, the halogen atom is replaced by a hydroxyl group, and the SOM is assigned to the carbon previously bonded to the halogen. This reaction is classified as general oxidative dehalogenation. In the second type, an epoxide is formed as a stable intermediate, in which case both carbon atoms forming the epoxide ring are labeled as SOMs. This reaction is referred to as epoxide-forming oxidative dehalogenation. Examples of both types are shown in Figure 10, panels F–H.

**Figure 10:**
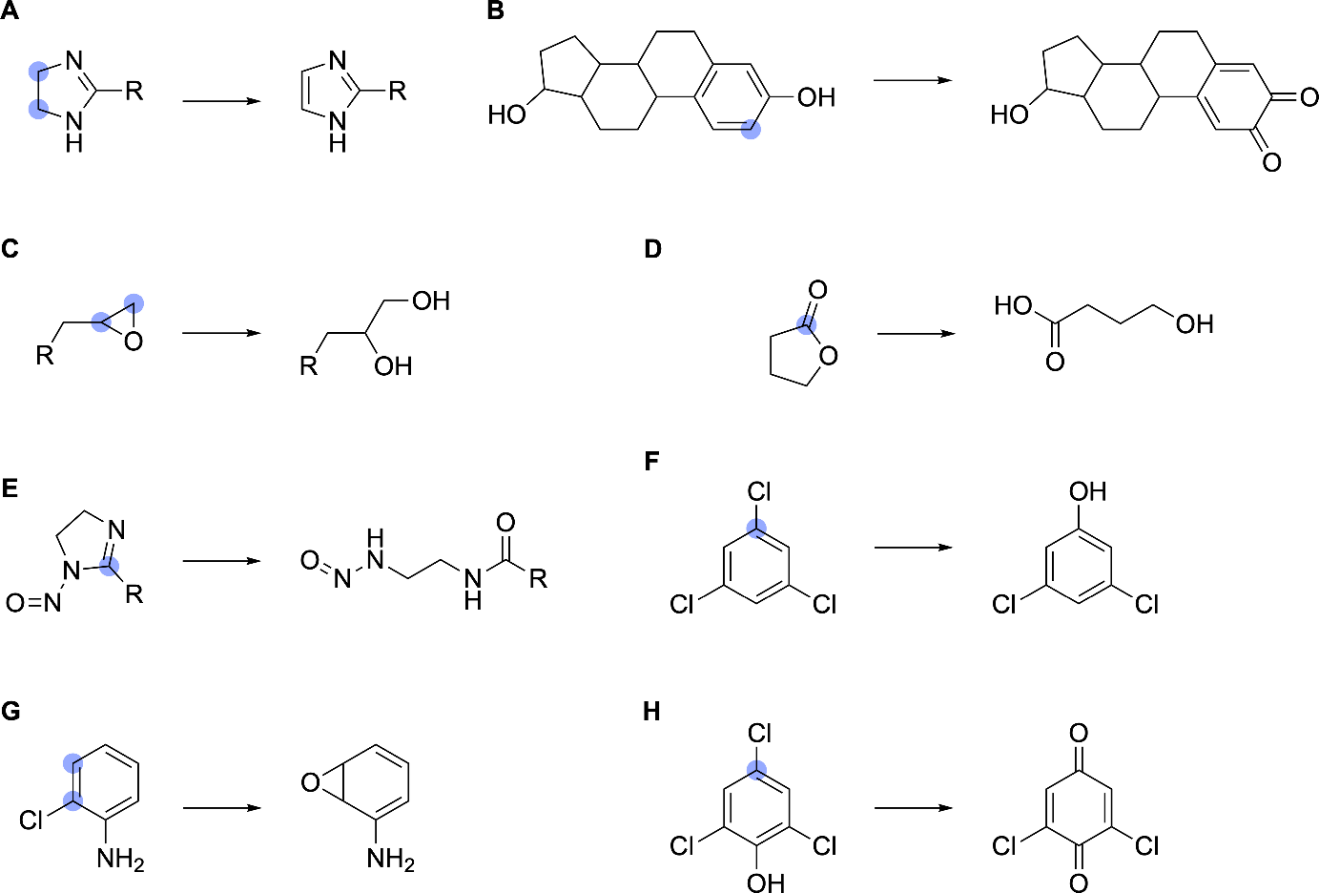
Examples of complex reaction handling. SOMs are denoted by blue circles. (A) Dehydrogenation of an imidazoline functional group, managed via subgraph isomorphism mapping. (B) Oxidation of a phenol functional group to 1,2-benzoquinone. This annotation requires MCS mapping. (C) Hydrolysis of an oxacyclopropane ring. (D) Hydrolysis of butyrolactone, which requires separate annotation since lactones are not recognized by the (thio)­ester hydrolysis function of the elimination handler. (E) Heterocyclic ring opening. (F–H) Three representative oxidative dehalogenation reactions.

The complex reaction annotator is particularly useful for metabolic transformations involving redox reactions, multiple bond changes, ring-opening reactions, and structural reorganizations that do not conform to simple addition or elimination patterns.

### 3.3 Output

The annotation pipeline returns the identified SOMs as a list of atom indices, the applied annotation rule, and the processing time for each reaction. Atoms that are topologically equivalent to the identified SOMs, such as those found in symmetrical environments like para-disubstituted aromatics, are also annotated during a postprocessing step. This correcting step is intended to account for ambiguity in atom mapping caused by molecular symmetry, particularly in cases where MCS-based mapping is applied. AutoSOM generates the output in multiple formats: a CSV file summarizing the results and three Structure Data (SD) files. These SD files include (i) one containing the annotated substrates, (ii) one containing the corresponding metabolites, and (iii) a consolidated file in which each unique substrate appears only once, with all its annotated SOMs merged into a single entry.

## 4 Results and Discussion

We begin this section by defining our evaluation metrics. Next, we assess AutoSOM’s labeling accuracy in detail using the MetaQSAR database. To demonstrate its versatility across different data sources, we evaluate its performance on both a proprietary, hand-curated, high-quality data set and a public data set. We then explore AutoSOM’s potential to leverage dormant metabolic data by comparing the predictive accuracy of two SOM-prediction models trained with expert-labeled and automatically labeled data. Finally, we conduct ablation studies to analyze the impact of individual annotation rules on labeling accuracy and discuss key practical considerations and limitations of AutoSOM.

### 4.1 Evaluation Metrics

We assess AutoSOM’s performance using three metrics: the percentage of full matches, partial matches, and neighbor matches. Each metric represents the proportion of correctly identified SOMs relative to the total number of reactions assigned to the category being evaluated (i.e., the entire data set, a general annotation rule, or a specific annotation rule). Full matches occur when all SOMs in a reaction are correctly identified, with no false positives and no false negatives. Partial matches occur when at least one SOM is correctly annotated, but the prediction includes either a false negative (a missing SOM) or a false positive (an incorrectly predicted SOM). Note that, in most cases, a metabolic reaction is associated with a single SOM. However, there are reactions—such as the epoxidation of alkenes—in which multiple atoms are labeled as SOMs. Neighbor matches occur when no SOM was correctly predicted, but the correct SOM is a direct neighbor of a predicted SOM. Illustrative examples are presented in Figure 11.

**Figure 11:**
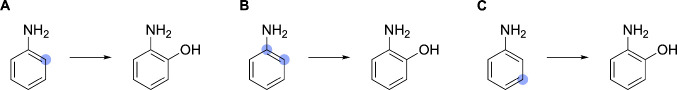
Visualization of the evaluation metrics, demonstrated using the ortho-hydroxylation of aniline. Predicted SOMs are indicated in blue. (A) Full match: the SOM is correctly identified, with no false positives and no false negatives. (B) Partial match: the SOM is correctly annotated, but the prediction includes a false positive. (C) Neighbor match: the correct SOM was not found, but the correct SOM is a direct neighbor of the predicted SOM.

### 4.2 Evaluating Labeling Accuracy on the MetaQSAR Data Set

#### 4.2.1 MetaQSAR Data Set

The MetaQSAR data set is a collection of more than 6000 mammalian metabolic biotransformations of xenobiotics. For this study, we used the October 2023 version and applied a rigorous curation process, a detailed description of which is provided in the Supporting Information. The preprocessed data set contains 5715 individual reactions and 2711 unique substrates (see Supporting Information, Section S2).

#### 4.2.2 Results

A total of 5708 reactions (99.9%) passed the timeout filter. Among these, the proportion of full matches is 93.6%, partial matches account for 2.6%, and neighbor matches make up 2.4%. The distribution of reactions across rule categories—addition, elimination, complex—along with their respective accuracy, is presented in Table 1. A detailed breakdown of AutoSOM’s performance across single annotation rules is presented in Table 2.

**Table 1:** Annotation Accuracy of AutoSOM on the MetaQSAR Data Set Per Annotation Rule Category

Rule category	Number of reactions	Full match %	Partial match %	Neighbor match %
All^a^	5708	93.6	2.6	2.4
Addition	3245	99.6	0.0	0.1
Elimination	1076	94.7	0.6	1.3
Complex	1387	78.8	10.2	8.6
Timeout filter	7	–	–	–
Unknown	0	–	–	–

a All reactions that passed the default timeout filter (20 s).

**Table 2:** Annotation Accuracy of AutoSOM on the MetaQSAR Data Set Per Annotation Rule

Rule category	Specific rule	Number of reactions	Full match %	Partial match %	Neighbor match %
Addition	General addition	2888	99.9	0.0	0.0
	Glutathione conjugation	347	96.8	0.0	0.3
	Carnitine conjugation	10	100.0	0.0	0.0
Elimination	General elimination	745	95.7	0.8	1.7
	(Thio)ester hydrolysis	274	95.6	0.0	0.4
	Hydrolysis of esters of inorganic acids	57	84.6	0.0	0.0
Complex	Oxidative dehalogenation	60	100.0	0.0	0.0
	Subgraph isomorphism mapping	413	96.1	3.9	0.0
	MCS mapping	778	67.7	14.4	14.3
	Oxacyclopropane hydrolysis	37	78.4	21.6	0.0
	Lactone hydrolysis	12	100.0	0.0	0.0
	Other heterocyclic ring hydrolysis	87	78.2	5.7	9.2
Timeout filter		7	–	–	–
Unknown		0	–	–	–

AutoSOM achieved near-perfect labeling accuracy for addition reactions, with a full match proportion of 99.6%. For elimination reactions, AutoSOM delivered strong performance, with a full match proportion of 94.7%. In the case of complex reactions, AutoSOM performed reasonably well, achieving a full match accuracy of 78.8% and a partial match accuracy of 10.2%. Besides the seven reactions that were filtered out by the timeout functionality, there were no reactions for which AutoSOM did not assign any SOM (“unknown” rule).

While the performance of AutoSOM on addition and elimination reactions—which constitute the majority of reactions—is likely sufficient for downstream applications (see Section 4.5), human annotators can further improve annotation accuracy by manually reviewing some of the automatically annotated reactions. The advantages of using AutoSOM as a preannotation tool for this task are 2-fold: First, rather than having to review all the data, human annotators would only need to focus on reactions in the complex and unknown categories. Second, for complex reactions, the proximity of incorrect annotations to the correct ones, as reflected by the 8.6% neighbor matches, facilitates the identification of correct atoms by reviewing only the predicted atoms and their direct neighbors.

### 4.3 Comparison of AutoSOM with Baseline Approaches

#### 4.3.1 Experimental Setup

We compare the performance of AutoSOM against three baseline approaches. The first baseline uses the MCS between the substrate and metabolite to generate AAMs. The second baseline leverages RXNMapper,^25^ a transformer-based AAM generator. The third baseline uses LocalMapper,^42^ a model that learns AAMs from chemist-labeled reactions via human-in-the-loop machine learning. After obtaining AAMs, SOMs are assigned to the substrate atoms experiencing topological changes. The MCS-based baseline was chosen for its alignment with existing automated SOM annotation methods.^10,23,24^ RXNMapper and LocalMapper were included to assess the scope and limitations of machine-learning-based AAM methods, which are trained on general organic reactions and may struggle with metabolic reaction data. In both cases, predicted SOMs were compared to ground-truth labels from the MetaQSAR database.

#### 4.3.2 Results

The MCS baseline achieved a full match rate of 66.4%, a partial match rate of 11.7%, and a neighbor match rate of 10.9%. RXNMapper attained full, partial, and neighbor match rates of 48.5%, 33.9%, and 16.6%, respectively. The LocalMapper baseline yielded corresponding match rates of 66.1%, 13.4%, and 18.7%, respectively. We hypothesize that the superior performance of the MCS-based and LocalMapper baselines compared to RXNMapper stems from the tendency of the latter to produce incorrect AAMs for metabolic reactions, leading to inaccurate SOM annotations (see Section 2.6, Figure 3).

Overall, all baselines performed substantially worse than AutoSOM, which achieved full, partial, and neighbor match rates of 93.6%, 2.6%, and 2.4%, respectively. This performance gap is expected, given that our ground truth consists of expert-labeled, mechanistically informed SOMs, a standard that traditional baselines were not designed to meet.

This comparison, far from dismissing the accuracy of RXNMapper and LocalMapper in generating AAMs for stoichiometrically balanced organic reactions, underscores the novelty of AutoSOM’s annotation pipeline. Notably, AutoSOM remains the only open-source method capable of producing chemically meaningful SOM annotations when applied to stoichiometrically unbalanced metabolic biotransformation records.

### 4.4 Evaluating Labeling Accuracy on an External, Public Data Set

#### 4.4.1 MetaTrans Data Set

The MetaTrans data set^47,48^ is a publicly available collection of 11,670 human metabolic biotransformations of endogenous and xenobiotic compounds. It has been compiled from multiple sources, including DrugBank (version 5.1.5),^49^ the Human Metabolome Database (version 4.0),^50^ HumanCyc from MetaCyc (version 23.0),^51^ Recon3D (version 3.01),^52^ the biotransformation database of BioTransformer,^53^ and the reaction rules from SyGMa.^54^ Note that the MetaTrans data set does not contain ground truth SOM annotations.

#### 4.4.2 Distribution of Annotation Rules

In the absence of ground-truth labels, we begin our evaluation by comparing the distribution of annotation rules applied in MetaTrans with those in the MetaQSAR data set (see Table 3).

**Table 3:** Distribution of Annotation Rule Categories Across Data Sets

Rule category	MetaQSAR [%]	MetaTrans [%]
Addition	56.8	46.4
Elimination	18.8	22.1
Complex	24.2	46.4
Timeout filter	0.0	0.0
Unknown	0.0	0.0

The proportion of addition reactions identified by AutoSOM was substantially lower in MetaTrans (31.4%) than in MetaQSAR (56.8%). This discrepancy appears to be partially offset by a higher proportion of complex reactions in MetaTrans (46.4%) compared to MetaQSAR (24.2%). Meanwhile, the proportions of elimination reactions were comparable between the two data sets, with 18.8% in MetaQSAR and 22.1% in MetaTrans.

Since complex reactions typically exhibit lower annotation accuracy, these results suggest that the SOM labels generated by AutoSOM for the MetaTrans data set may include a higher rate of inaccurate or incorrect annotations compared to those generated from MetaQSAR. Notably, a high degree of automated data processing was employed in compiling MetaTrans, whereas all MetaQSAR data was manually compiled and curated by experts. We, therefore, hypothesize that a substantial proportion of reactions labeled as complex in MetaTrans may actually be mislabeled due to limitations of AutoSOM in accounting for multistep reactions and data records representing multiple reactions with a single substrate-metabolite pair. Evidence supporting these considerations is presented in the following paragraph.

#### 4.4.3 Labeling Accuracy

Due to a lack of ground truth SOM annotations in the MetaTrans data set, the evaluation of AutoSOM’s labeling performance was performed via a manual analysis of a subset of reactions. To ensure a statistically meaningful evaluation, we selected a random sample of 140 reactions, which we refer to as the MetaTrans subset. This sample size was chosen to estimate the true labeling accuracy of AutoSOM on the entire MetaTrans data set with a 95% confidence level and a 5% margin of error. The calculation is based on a conservative prior accuracy of 90%, slightly below the observed 93.6% full match accuracy on the MetaQSAR data set (see Section 4.2). Details of the sample size determination are provided in the Supporting Information, Section S3. We categorized each annotation into three main groups: correct, incorrect, and inapplicable. The latter category includes cases where AutoSOM cannot reasonably be expected to provide an accurate annotation, such as multistep reactions, which lie outside its applicability domain. A summary of the evaluation results is provided in Table 4. A comprehensive list of evaluated substrate-metabolite pairs, along with their automatically annotated SOMs and evaluation results, is available in the Supporting Information. Representative examples of incorrect and inapplicable reactions are discussed in the Supporting Information, Section S3.

**Table 4:** Annotation Accuracy of AutoSOM on the MetaTrans Subset

		Correct		Incorrect		Inapplicable	
Rule category	Number of reactions	Number	Percentage	Number	Percentage	Number	Percentage
Addition	44	42	95.5	0	0.0	2	4.5
Elimination	31	31	100.0	0	0.0	0	0.0
Complex	65	40	61.5	10	15.4	15	23.1
Timeout filter	7	–	–	–	–	–	–
Unknown	0	–	–	–	–	–	–

The results presented in Table 4 indicate that the labeling accuracy of AutoSOM on the MetaTrans subset is consistent with its accuracy on the MetaQSAR data set, provided that inapplicable reactions are excluded. For each annotation rule category, we report the total number of reactions assigned to that category by AutoSOM, along with the number and percentage of correct, incorrect, and inapplicable annotations.

- **Addition reactions.** The percentage of correctly annotated reactions is 95.5%, which is slightly lower than the full-match accuracy on addition reactions in the MetaQSAR data set (99.6%). However, considering that 4.5% of the addition reactions were deemed inapplicable, this is a satisfactory result.
- **Elimination reactions.** The correct annotation rate is 100%, which is higher than the full-match accuracy on elimination reactions in the MetaQSAR data set (94.7%).
- **Complex reactions.** The correct annotation rate is 61.5%, which is lower than the accuracy of the MetaQSAR data set for complex reactions (78.8%). However, 23.1% of all complex reactions were classified as inapplicable, which almost exactly bridges the performance gap between the two data sets. This relatively high proportion of inapplicable cases supports the suggestion that the MetaTrans data set contains more multistep reactions and data records representing multiple reactions with a single substrate-metabolite pair compared to the MetaQSAR data set, which was specifically curated to minimize these cases.

Overall, across all reaction categories, there were only 10 incorrect annotations (7.1%) and 17 inapplicable reactions (12.1%).

### 4.5 Evaluating AutoSOM’s Potential for Leveraging Dormant Metabolic Data

#### 4.5.1 Experimental Setup

In this experiment, we explored whether AutoSOM’s automatically generated labels are sufficiently accurate to train SOM predictors that perform on par with those trained on expert-labeled data. To this end, we randomly split the MetaQSAR data set into training (80%) and test (20%) sets. We then created two versions of the training set, differing only in their labels: an expert-derived training set, containing the original SOM labels from the MetaQSAR database, and an automatically generated training set, containing SOM labels predicted by AutoSOM.

Using these two training sets, we retrained two established SOM predictors, FAME3R^55^ and aweSOM,^56^ resulting in four models. FAME3R is a Random Forest (RF)-based SOM prediction model and a reimplementation of the CDK-based^57^ FAME3 ^13^ using CDPKit.^58^ It was recently employed in a study exploring active learning as a strategy to advance SOM prediction.^55^ aweSOM is a GNN-based SOM prediction model. The performance of FAME3, FAME3R, and aweSOM is comparable, with the latter providing aleatoric and epistemic uncertainty estimates alongside its SOM predictions. Detailed descriptions of aweSOM’s and FAME3R’s hyperparameters are presented in the Supporting Information, Section S5. All models were evaluated using the same test set, which contains the expert-derived SOM labels from the MetaQSAR database. The experimental setup is depicted in Figure 12.

**Figure 12:**
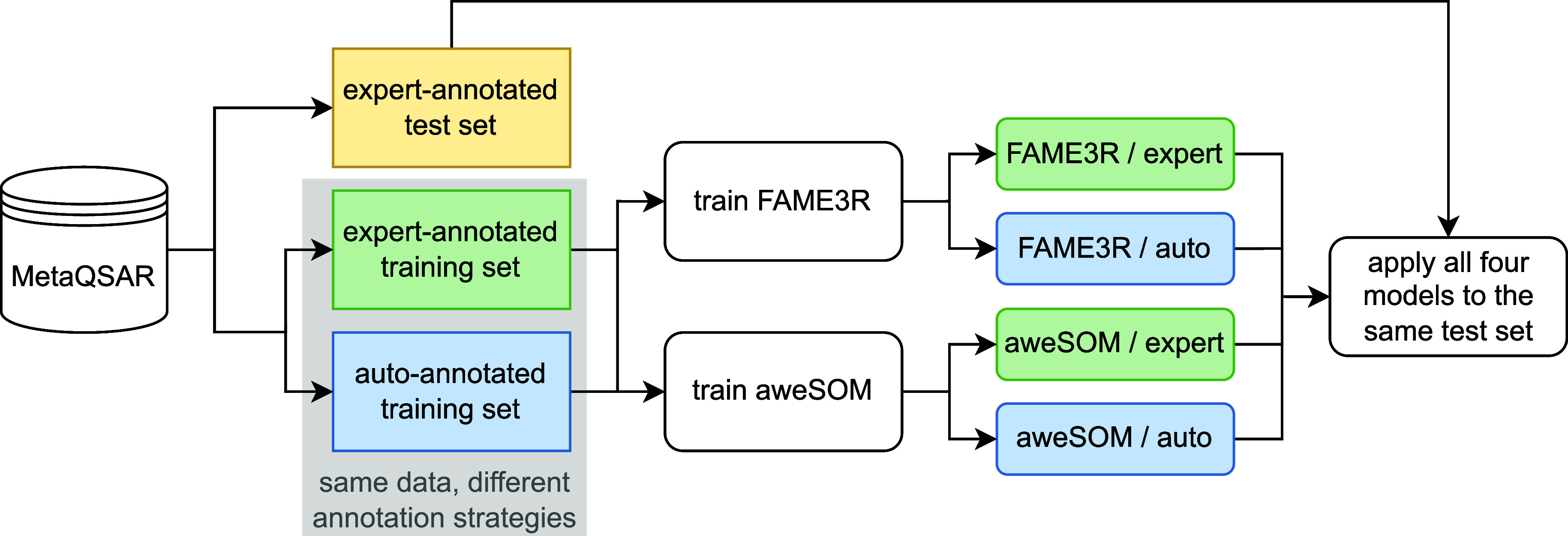
Experimental setup for training and evaluating established SOM-predictors FAME3R and aweSOM trained on expert-derived and automatically labeled data.

#### 4.5.2 Results

The results, summarized in Table 5, show that both FAME3R and aweSOM, when trained on AutoSOM-generated labels, achieve performance levels comparable to those obtained when using expert-labeled data. These findings confirm that AutoSOM is sufficiently accurate for the automated SOM annotation of biotransformation data, enabling the mobilization of dormant metabolism data for SOM predictor development.

**Table 5:** Comparison of SOM-Prediction Models Trained on Expert vs Automatically Annotated Data from the MetaQSAR Database^a^ ^,^ ^b^

	FAME3R		aweSOM	
	Expert	Auto	Expert	Auto
ROC-AUC	0.91 ± 0.01	0.90 ± 0.01	0.91 ± 0.01	0.90 ± 0.01
PR-AUC	0.59 ± 0.01	0.55 ± 0.02	0.57 ± 0.01	0.54 ± 0.01
F1-score	0.56 ± 0.01	0.54 ± 0.01	0.56 ± 0.01	0.54 ± 0.01
MCC	0.51 ± 0.01	0.49 ± 0.01	0.51 ± 0.01	0.48 ± 0.01
Precision	0.56 ± 0.01	0.54 ± 0.01	0.52 ± 0.01	0.47 ± 0.01
Recall	0.56 ± 0.02	0.54 ± 0.02	0.61 ± 0.01	0.63 ± 0.01
TOP-2	0.80 ± 0.01	0.79 ± 0.01	0.82 ± 0.01	0.79 ± 0.01

a ROC-AUC: area under the receiver operating characteristics curve, PR-AUC: area under the precision-recall curve, MCC: Matthews correlation coefficient, TOP-2: proportion of substrates where at least one true SOM is among the top-2 predictions ranked by decreasing predicted SOM probability.

b Mean and standard deviation were calculated from 1000 bootstrap samples, each drawn with replacement from the test set, containing 538 molecules.

### 4.6 Ablation Study

#### 4.6.1 Experimental Setup

In this experiment, we examined how labeling accuracy improves as a function of the number of annotation rules. Specifically, we plotted AutoSOM’s labeling accuracy on the MetaQSAR data set as a function of an incrementally growing set of annotation rules (see Figure 13). The rules were ordered based on the number of reactions they were applied to (numbers in parentheses), with the most common rule applied first and the least common applied last.

**Figure 13:**
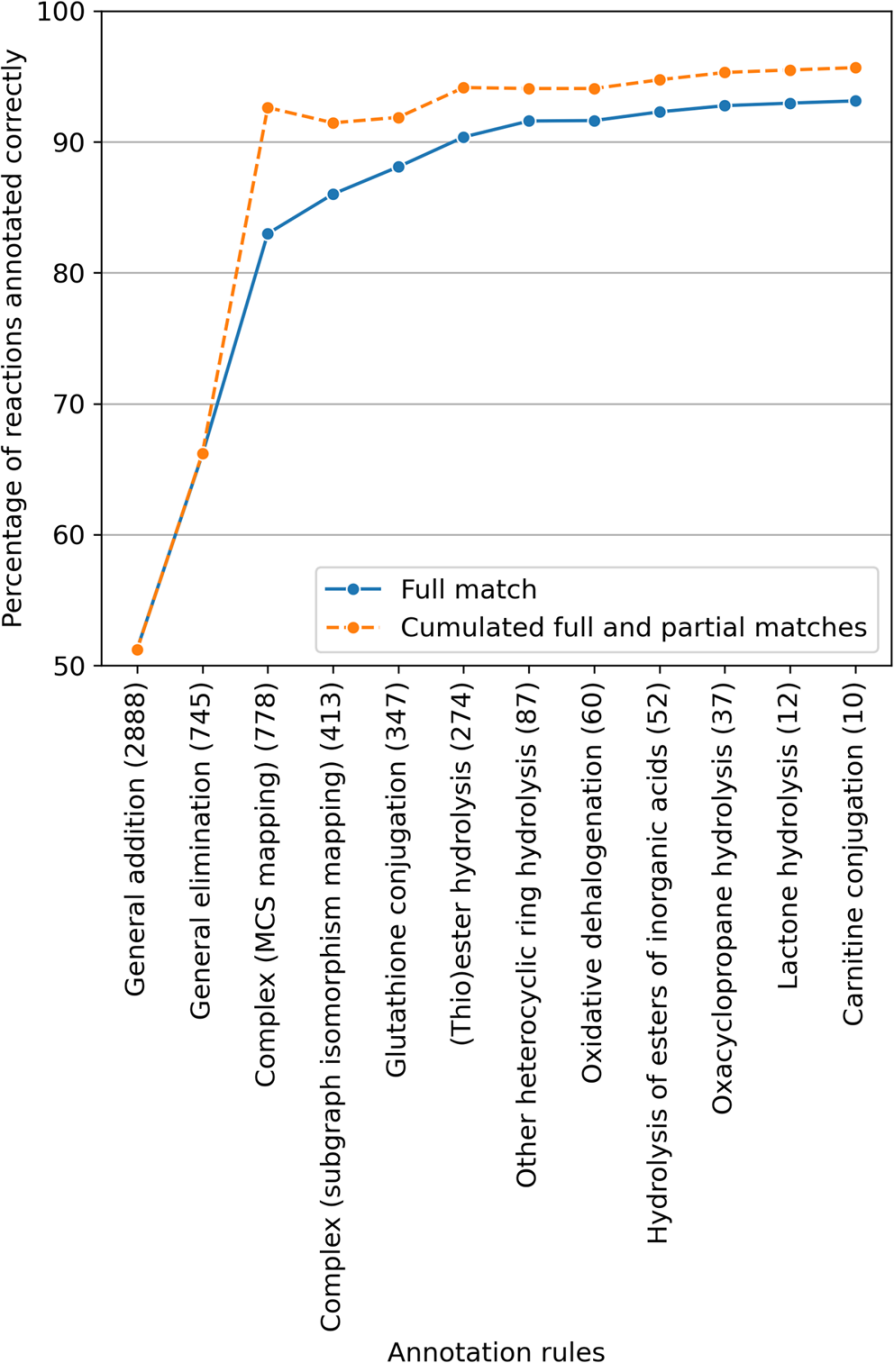
Labeling accuracy of AutoSOM on the MetaQSAR data set as a function of the number of annotation rules. Rules were added incrementally, starting with the most frequently applied rule and ending with the least common one.

#### 4.6.2 Results

As shown in Figure 13, applying only a few key rules is sufficient to achieve good annotation performance. The performance begins to plateau after the first six most important rules (general addition reaction, general elimination reaction, MCS mapping, subgraph isomorphism mapping, glutathione conjugation reaction, and ester hydrolysis reaction). Further improvements in accuracy require the application of increasingly specific rules. This suggests that, in theory, a near-perfect annotator could be designed, but doing so would require exponentially greater effort, as additional rules would need to be highly specialized and apply to progressively fewer cases.

### 4.7 Practical Considerations and Limitations

#### 4.7.1 Runtime

The main runtime bottleneck in AutoSOM lies in the substructure matching steps used throughout the algorithm. Finding the maximum common substructure between two graphs has a worst-case complexity of *O*(2^*n*^). However, RDKit employs heuristics such as branch-and-bound pruning, atom/bond constraints, and early stopping criteria, making it efficient for small to medium-sized molecules. For larger molecules, runtime can increase significantly due to the combinatorial nature of subgraph matching. To address this, AutoSOM implements a timeout parameter that limits the maximum allowed time per annotation trial. Excluding the seven reactions that timed out after 20 s, the average runtime per reaction is approximately 52 ms on a system equipped with an AMD Ryzen 9 7950X 16-core CPU.

As detailed in Section 3, AutoSOM employs RDKit’s maximum common substructure matching algorithm with varying atom- and bond-matching constraints depending on the annotation rule. The most efficient parameters are used for additions and eliminations, with the most computationally intensive criteria applied to complex transformations. Figure 14 illustrates the relationship between increasing matching complexity and the average runtime per annotated substrate.

**Figure 14:**
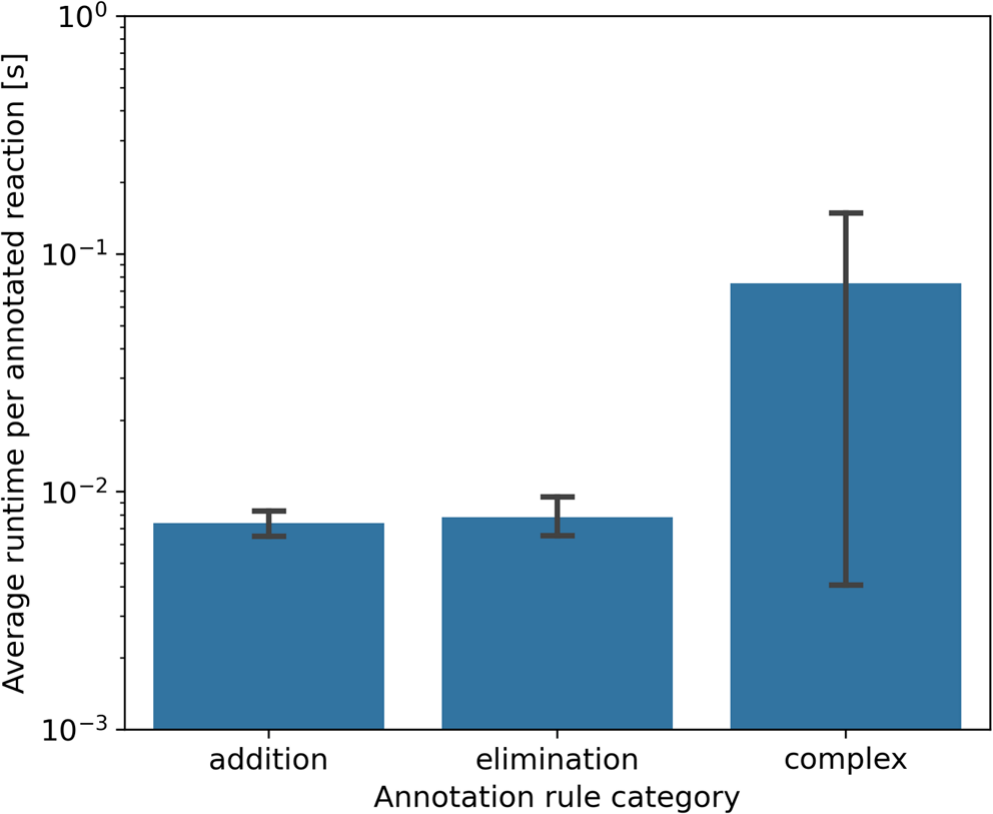
Bar plot showing the average runtime per annotated substrate for different annotation rule categories, with error bars representing the 95% confidence intervals. As matching complexity increases, runtime also increases. Simpler categories, such as addition and elimination, exhibit shorter runtimes, while complex reactions sometimes require more time.

#### 4.7.2 Using AutoSOM to Validate Existing Annotations

Curating metabolism data requires expert knowledge and is susceptible to errors. Common mistakes include incorrectly drawing the structure of either the substrate or metabolite, or misannotating atoms, especially in large molecules with similar but nonequivalent functional groups. AutoSOM can assist in validating existing annotations by identifying discrepancies between manual and automated annotations. Instead of reviewing all entries, curators can focus only on cases where the annotations differ, significantly reducing the effort required for quality control. Examples of incorrect database entries that could easily be identified using AutoSOM are provided in Figure 15 and Figure 16.

**Figure 15:**
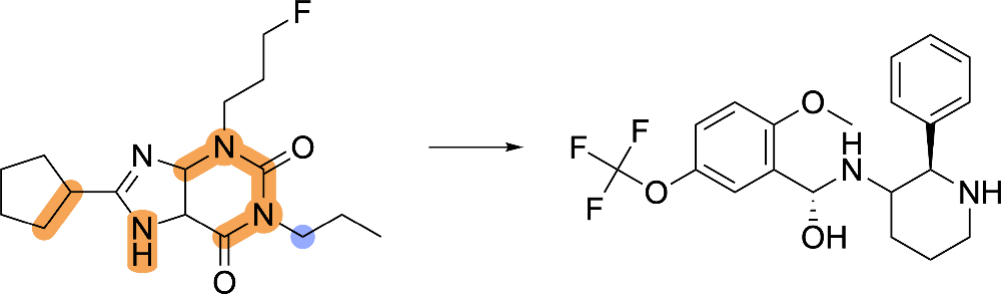
Example of an erroneous database entry detected and corrected using AutoSOM. In this case, the substrate was incorrectly linked to an implausible metabolite. AutoSOM helped flag the error by highlighting discrepancies between expert-annotated SOMs and automatically generated annotations. Only entries where the two annotations differed required manual review. Expert-annotated SOMs are shown in blue, while AutoSOM-generated annotations are shown in orange.

**Figure 16:**
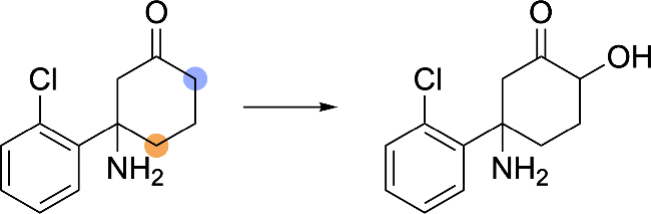
Example of an erroneous database entry detected and corrected using AutoSOM. In this case, the SOM was assigned to the wrong atom. AutoSOM helped flag the error by highlighting discrepancies between expert-annotated SOMs and automatically generated annotations. Only entries where the two annotations differed required manual review. The incorrect, expert-annotated SOM is shown in orange, while the correct, AutoSOM-generated annotation is shown in blue.

#### 4.7.3 Expanding to Multistep Reactions

Owed to the complexities in measuring and interpreting biotransformation data, public and industry data typically include multistep reactions (see Figure 17 for an example). Currently, AutoSOM supports only single-step metabolic reactions. We envisage developing strategies to infer the likely intermediates of multistep reactions by leveraging metabolite structure prediction tools such as Biotransformer,^53^ GLORYx,^17^ or Meteor.^19^ This could eventually allow the decomposition of complex reactions into individual steps that AutoSOM can accurately annotate.

**Figure 17:**
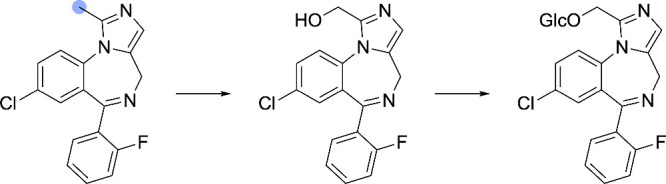
Example of a multistep reaction. Here, midazolam undergoes two sequential transformations: hydroxylation followed by glucuronidation of the intermediate metabolite. The hydroxylated intermediate would be absent from the database. Such multistep entries may result from the instability of intermediates, which prevents their isolation and characterization, or from curation errors. AutoSOM is not explicitly designed to handle multistep reactions and cannot flag them. However, in simple cases—such as this one—it may still incidentally generate a correct annotation. The blue circle indicates the correctly annotated SOM.

#### 4.7.4 Handling Metabolites Resulting from Multiple Reactions

AutoSOM is not designed to annotate substrate-metabolite pairs where more than one reaction has occurred. Consider, for example, two independent elimination reactions that remove R1 and R2 from a core structure, generating three possible metabolites: “R1-core,” “R2-core,” and “core”. While AutoSOM accurately annotates the reactions leading to the first two metabolites, it is not explicitly designed to handle the third case. Nevertheless, for most addition and elimination reactions, which are the predominant types in metabolic pathways, AutoSOM often incidentally annotates these cases correctly. An example is provided and discussed in Figure 18. Future developments will aim to systematically address multireaction scenarios, including complex reactions.

**Figure 18:**
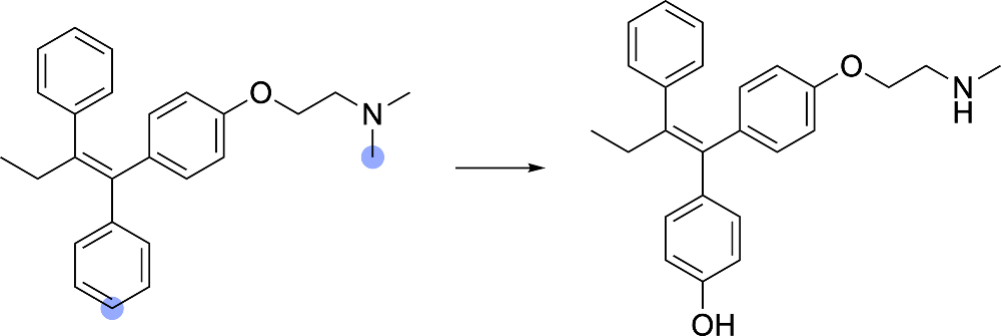
Example of a multireaction database entry. In this case, tamoxifen undergoes both aromatic hydroxylation and N-demethylation. Although AutoSOM is not explicitly designed to handle multireaction cases, it often incidentally produces correct annotations for simple addition and/or elimination reactions, as demonstrated in this example. The SOMs are marked by blue circles.

#### 4.7.5 Handling Chiral Inversions

One current limitation of AutoSOM is its lack of support for reactions involving changes in chirality, such as stereochemical inversion. We acknowledge the significance of this issue and plan to extend AutoSOM’s capabilities to accurately process chiral centers in future versions.

#### 4.7.6 Enhancing AutoSOM with Community Contributions

When developing AutoSOM, our goal was to maximize reaction coverage while minimizing the number of annotation rules. In its current state, AutoSOM provides rules for the most widely occurring reaction types in the MetaQSAR database. A general rule may incidentally annotate uncommon reactions correctly, but we do not guarantee accuracy for those exotic reaction types. By making AutoSOM open-source, our goal is to enable future users to refine and expand its rule set, improving coverage for specialized reaction types relevant to their applications.

#### 4.7.7 Addressing Subjectivity in Annotation Rules

The annotation rules in AutoSOM were primarily inspired by our observations of SOM labels in the MetaQSAR data set. We acknowledge both our subjectivity and that of the MetaQSAR curators in the interpretation of SOM annotations. However, as an open-source tool, AutoSOM invites scrutiny and collaboration from the metabolism community. We hope that ongoing discussions and contributions will help refine and expand AutoSOM, making it as universally applicable as possible.

#### 4.7.8 Enabling Federated Learning Approaches

Consortia across academia and industry can leverage AutoSOM to ensure consistent labeling of metabolism data, facilitating federated machine learning. A major challenge in developing SOM prediction tools, beyond the scarcity of annotated training data, is the heterogeneity of existing annotations. This inconsistency hampers machine learning models when applied to data sets from diverse sources. Addressing this issue is particularly challenging due to concerns about data confidentiality. By enabling multiple institutions to collaboratively annotate metabolism data in a standardized manner without sharing proprietary data sets, AutoSOM can help expand the volume of high-quality training data available for SOM prediction. Future advancements could focus on optimizing SOM predictors for distributed learning environments, integrating differential privacy techniques, and establishing standardized protocols for cross-institutional model updates.

## 5 Conclusions

In this work, we developed AutoSOM, the first open-source software capable of automatically extracting chemically meaningful SOMs from substrate-metabolite pairs with high accuracy. AutoSOM employs a fully explainable, rule-based annotation pipeline derived from expert-curated biotransformation data.

To evaluate AutoSOM’s performance, we rigorously compared the automatically derived annotations to expert-annotated ground truth labels in MetaQSAR and tested its runtime. Our results demonstrate that AutoSOM is accurate and fast, achieving over 90% labeling accuracy on a diverse validation set of more than 5000 reactions within minutes.

Additionally, we analyzed AutoSOM’s performance on MetaTrans, a public data set for which we can provide full annotation results, demonstrating that AutoSOM performs consistently well across data sets.

We showcased AutoSOM’s potential to advance the development of in silico models by leveraging dormant biotransformation data. Specifically, we trained two distinct SOM predictors on automatically labeled data and found that these models achieve comparable predictive accuracy to those trained on expert-derived data.

SOM predictors are essential not only as standalone models but also as integral components of metabolite structure prediction tools. AutoSOM provides a scalable solution for increasing the quantity of available annotated training data, thereby contributing to the improvement of predictive performance in future metabolism prediction tools.

AutoSOM can serve as a quality control tool by identifying structural inconsistencies and annotation errors in existing data sets, reducing the need for exhaustive manual review. It also facilitates the integration of multiple data sources by ensuring consistent annotation across data sets in a cost-effective and efficient manner.

By making AutoSOM open-source, we encourage community contributions to refine its annotation rules and further enhance its accuracy. By addressing both the quantity and homogeneity of data available to train metabolism prediction tools, we aim to advance the field of computational metabolism prediction.

## Supplementary Material





## Data Availability

The source code of the software presented in this work is available at https://github.com/molinfo-vienna/AutoSOM.

## Notes

Document detailing the software used in this work, the composition of the data set, the data preprocessing steps, and a discussion of representative examples of annotated substrate-metabolite pairs (PDF)
Text file containing the complete list of evaluated MetaTrans substrate-metabolite pairs (TXT)
