# Supplementary material for: Automated Annotation of Sites of Metabolism from Biotransformation Data
Source: J Chem Inf Model. 2025 Jun 17;65(13):7065–80. doi: 10.1021/acs.jcim.5c00819 (PMC12264948; doi:10.1021/acs.jcim.5c00819)
Supplement: Supplementary file 1 [file ci5c00819_si_001.pdf]

Automated Annotation of Sites of Metabolism from Biotransformation Data

Roxane Jacob,[†,‡,‖] Angelica Mazzolari,[¶] and Johannes Kirchmair[*,†,§]

†*Department of Pharmaceutical Sciences, Division of Pharmaceutical Chemistry, Faculty of Life Sciences, University of Vienna, Josef-Holaubek-Platz 2, 1090 Vienna, Austria*

‡*Christian Doppler Laboratory for Molecular Informatics in the Biosciences, Department of Pharmaceutical Sciences, University of Vienna, Josef-Holaubek-Platz 2, 1090 Vienna, Austria*

¶*Dipartimento di Scienze Farmaceutiche, Universita degli Studi di Milano, I-20133 Milano, Italy*

§*Christian Doppler Laboratory for Molecular Informatics in the Biosciences, Department for Pharmaceutical Sciences, University of Vienna, Josef-Holaubek-Platz 2, 1090 Vienna, Austria*

‖*Vienna Doctoral School of Pharmaceutical, Nutritional and Sport Sciences, University of Vienna, Josef-Holaubek-Platz 2, 1090 Vienna, Austria*

E-mail: johannes.kirchmair@univie.ac.at

S1 Software

AutoSOM is written using Python (3.11.0). Packages used by AutoSOM include NetworkX (3.4.2), NumPy (2.2.3), Pandas (2.2.3), and RDKit (2024.9.5). Data preprocessing, analysis and visualization was done in Python (3.11.0) using RDKit (2024.9.5), matplotlib (3.10.0), scikit-learn (1.6.1), and seaborn (0.13.2).

S2 MetaQSAR data set

The MetaQSAR data set is a proprietary collection comprising over 6,000 drug-like substrate-metabolite pairs spanning 101 reaction types. The reaction types are grouped into 21 reaction classes and 3 reaction main classes. A list of reaction classes as well as their relative frequency is presented in Table S1.

For this study, we used the version of October 2023[1] and applied a rigorous curation process consisting of the following steps:

1. Removed reactions involving compounds containing elements other than hydrogen, carbon, nitrogen, oxygen, sulfur, phosphorus, fluorine, chlorine, bromine, iodine, boron, silicon, or selenium.

2. Excluded Site-of-Metabolism (SOM) labels marked as "uncertain" by the MetaQSAR curators. If a reaction had no certain SOMs, we removed it from the data set.

3. Solved duplicate reactions (same substrate and metabolite identifiers): if all duplicates had identical SOM labels, we retained only one instance, if they had conflicting SOM labels, we removed all instances.

4. Removed entries where an International Chemical Indentifier (InChI) could not be computed.

5. Eliminated cases where different substrate identifiers mapped to the same InChI.

6. Removed entries where identical substrate-metabolite pairs (based on InChI) mapped to different pairs of substrate and metabolite identifiers.

Additionally, we corrected SOM labels where appropriate and extended SOM annotations to

topologically equivalent atoms within the same substrate. The preprocessed data set contains 5715 individual reactions and 2711 unique substrates.

Table S1: MetaQSAR classification of metabolic reactions.

Category	ID	Description	%*
Redox reactions	1	Oxidation of sp^3 hybridized carbon atoms	28.9
	2	Oxidation of sp and sp^2 hybridized carbon atoms	15.3
	3	Redox reactions between alcohols, aldehydes, ketones and carboxylic acids	3.8
	4	Various redox reactions of carbon atoms	1.1
	5	Redox reactions of tertiary amines	2.3
	6	Oxidation of secondary amines, oximes and nitroso etc.; reduction of nitro, nitroso and oximes etc.	3.1
	7	Oxidation to quinones or analogs; reduction of quinones and analogs	2.3
	8	Oxidation and reduction of sulfur atoms	2.7
	9	Redox reactions of other atoms	0.1
Hydrolysis and other reactions	10	Hydrolysis of esters, lactones and inorganic esters	6.1
	11	Hydrolysis of amides, lactams and peptides	2.9
	12	Epoxide hydration	0.5
	13	Other hydrolysis and hydration reactions; non-enzymatic eliminations and rearrangements	2.3
Conjugation reactions	14	Oxygen-glucuronidations and -glycosylations	11.2
	15	Nitrogen- and sulfur-glucuronidations; all other glycosylations	2.2
	16	Sulfonations	1.9
	17	Glutathione and RSH conjugations and their sequels; glutathione-mediated reductions	6.9
	18	Acetylations & acylations	1.5
	19	CoASH-ligation followed by amino acid conjugations or other sequels	1.3
	20	Methylations	1.4
	21	Other conjugations; transaminations	1.0

*The percentages presented in this table were computed after preprocessing.

S3 Examples of evaluation results on the MetaTrans data set

The MetaTrans dataset[2,3] is a publicly available collection of 11,670 human metabolic biotransformations involving both endogenous and xenobiotic compounds. We used this dataset as an external benchmark to evaluate AutoSOM.

Since the MetaTrans dataset does not include ground truth SOM annotations, we evaluated AutoSOM's labeling performance through manual analysis of a randomly selected subset of 140 reactions. The sample size was determined using:

$$n = \frac{Z^2 \cdot p \cdot (1 - p)}{E^2} \tag{1}$$

where Z represents critical value for the desired confidence level, p is the assumed prior accuracy, and E is the acceptable margin of error. Assuming a conservative prior accuracy of 90% (see Section 4.2), and setting the desired estimation accuracy to 95% confidence ($Z = 1.96$) with a 5% margin of error, we calculated a required sample size of 138.3, which was rounded up to 140.

Each annotation was classified into one of three categories: correct, incorrect, or inapplicable. The inapplicable category includes cases where AutoSOM cannot reasonably be expected to provide accurate annotations, such as multi-step reactions, which fall outside its applicability domain. A summary of the evaluation results is provided in the main body of this work.

The complete list of evaluated substrate-metabolite pairs, along with their corresponding SOMs, annotation rules, annotation times (in seconds), and evaluation results, is available in the attached .csv file. In this file, a value of zero represents an incorrect annotation, one

denotes a correct annotation, and two signifies an inapplicable annotation.

Below, we discuss representative examples of inapplicable and incorrect reactions.

Figure S1 presents an entry classified as inapplicable due to being both multi-step and involving multiple distinct reactions. The substrate, cholecalciferol (vitamin D), undergoes oxidation at three distinct positions, followed by glucuronidation and a secondary alcohol-to-ketone oxidation. The annotated SOMs are highlighted with blue circles. Although AutoSOM correctly identified all three aliphatic carbon atoms involved in the reaction, we consistently exclude such multi-step/multi-reaction entries from our evaluation, as AutoSOM is not specifically designed to handle these cases.

Figure S2 illustrates another example of a multi-reaction entry. The substrate, thymidine triphosphate, undergoes both dephosphorylation and conversion of thymine to cytosine, resulting in the formation of cytidine diphosphate.

Figure S3 presents an example of another type of "inapplicable" biotransformation: an epimerization reaction. Since AutoSOM does not currently support the processing of stereochemical information, epimerization reactions fall outside its scope of applicability. In those cases, AutoSOM returns an empty list of SOMs.

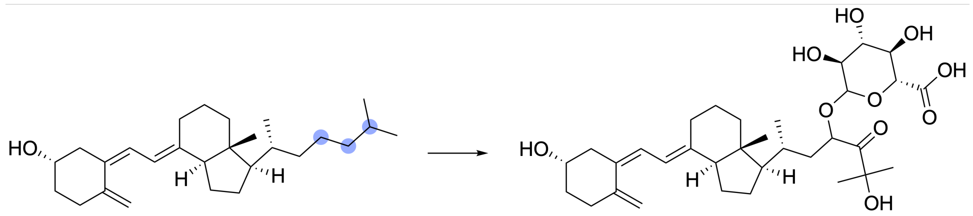

Figure S1: Example of an inapplicable reaction due to its multi-step nature and involvement of multiple distinct reactions. The substrate, cholecalciferol (vitamin D), undergoes oxidation at three distinct positions, followed by glucuronidation and a secondary alcohol-to-ketone conversion. Such cases fall outside the applicability domain of AutoSOM.

Figure S4 illustrates a representative example of an incorrect annotation by AutoSOM. The reaction involves the conversion of cyclic guanosine monophosphate to guanosine monophos-

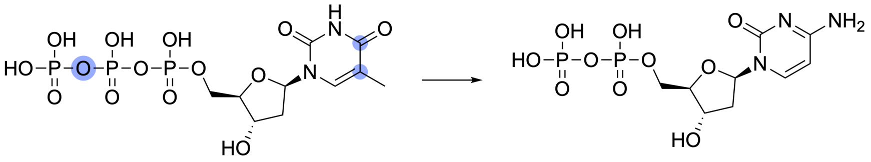

Figure S2: Example of a multi-reaction entry. The substrate, thymidine triphosphate, undergoes both dephosphorylation and conversion of thymine to cytosine, resulting in the formation of cytidine diphosphate. These reactions exceed AutoSOM's scope, as it is not designed to handle complex multi-step transformations.

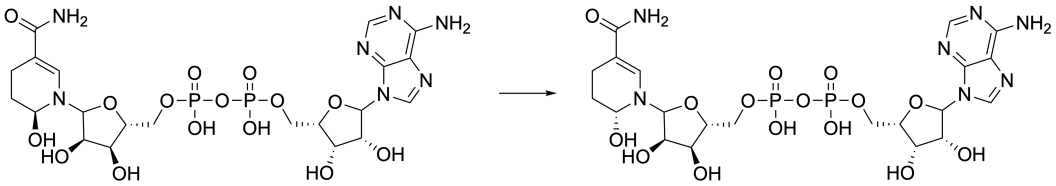

Figure S3: Example of an "inapplicable" reaction: epimerization. Since AutoSOM does not process stereochemical information, such reactions fall outside its scope of applicability.

phate. The SOM predicted by AutoSOM is highlighted with an orange circle, while the correct SOM is marked with a blue circle.

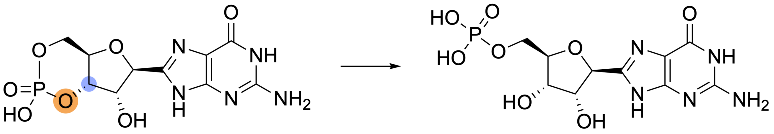

Figure S4: Example of an incorrectly annotated reaction by AutoSOM. The conversion from cyclic guanosine monophosphate to guanosine monophosphate is shown, where the automatically assigned SOM (highlighted in orange) differs from the correct SOM (highlighted in blue).

S4 Representative selection of annotation errors

In this section, we present representative examples of annotation errors to highlight current limitations of AutoSOM. These cases are intended to illustrate common challenges, but they by no means represent an exhaustive list of issues. Providing a comprehensive list of incorrect annotations is not feasible due to licensing restrictions associated with the MetaQSAR database. Nevertheless, these examples provide valuable insight into areas where

AutoSOM can be improved. We plan to address several of these issues in future versions and encourage contributions from the broader community—particularly institutions that benefit from AutoSOM—to help enhance the tool's overall performance.

Figure S5 illustrates a misannotation involving the elongation reaction of the alkyl chain on a carboxylic acid, which was incorrectly processed by the addition reaction handler. The predicted SOM (highlighted in orange) corresponds to the atom gaining a new neighbor. From the perspective of a Maximum Common Substructure (MCS)-based logic, this would be a reasonable prediction. However, MetaQSAR curators have annotated the carbonyl carbon atom as the true SOM (blue highlight) based on expert mechanistic insight.

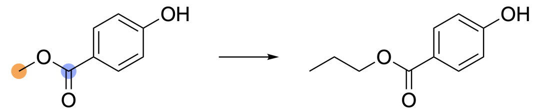

Figure S5: Erroneous annotation of the elongation reaction of the alkyl chain of a carboxylic acid. The predicted SOM (orange) corresponds to the atom gaining a new neighbor, which aligns with MCS-based logic, but differs from the expert-curated true SOM (blue).

Figure S6 illustrates a misannotation in a glutathione conjugation reaction. In this case, AutoSOM failed to correctly differentiate between the leftmost and rightmost carbon atoms in the substrate, ultimately selecting one at random. This error arises in substrates that exhibit near-symmetry, differing only in bond order—an aspect that current versions of AutoSOM do not yet handle robustly. To address this limitation, future versions will incorporate improved support for glutathione conjugation reactions by factoring in the valence states of candidate atoms, enabling more accurate SOM predictions in such edge cases.

Figure S7 illustrates a failed annotation of an N-dealkylation reaction. The current version of AutoSOM struggles with this reaction type, particularly when the reactions results in an N,N-dimethylamine moiety. The existing Atom-to-Atom-Mapping (AAM) strategy is unable to reliably identify which methyl group in the metabolite corresponds to which in the substrate. As a result, the annotation effectively becomes a coin toss, with only a 50:50

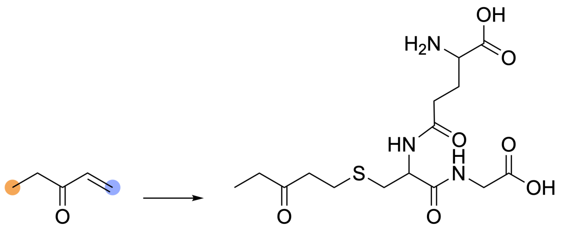

Figure S6: Incorrect annotation of a glutathione conjugation reaction where AutoSOM fails to distinguish between symmetrical carbon atoms in the substrate and selects one at random. This issue arises due to near-symmetry differing only by bond order, which future versions will address by considering atomic valence. The true SOM are shown is blue, whereas the predicted SOM is shown in orange.

chance of correctly assigning the true SOM.

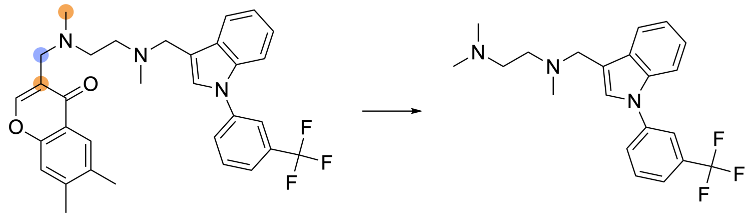

Figure S7: Failed annotation of an N-dealkylation reaction resulting in an N,N-dimethylamine moiety. The current AAM strategy cannot reliably identify the correct methyl group, resulting in a 50:50 chance of correct SOM assignment. The true SOM are shown is blue, whereas the predicted SOMs are shown in orange.

Figure S8 illustrates another failed annotation, involving an elimination reaction in the category of ester hydrolysis. This example is one of the few cases where the cleaved ester group is directly adjacent to another ester moiety. While the pipeline correctly identifies the cleavage event, it mistakenly selects the wrong carbonyl atom as the SOM. In the figure, the predicted SOM is indicated by an orange circle, whereas the true SOM is marked by a blue circle. Future versions of AutoSOM will include a correction to address this specific type of error.

Figures S9 and S10 present examples of incorrectly annotated reactions categorized as "complex." This category exhibits a lower success rate compared to addition and elimination reactions, largely because it encompasses a more diverse range of reaction types. The two figures highlight the two most common challenges encountered in this category: ring opening

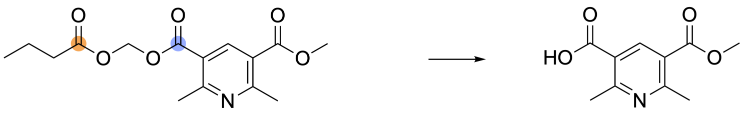

Figure S8: Misannotation of an ester hydrolysis elimination reaction where two adjacent ester moieties cause AutoSOM to select the wrong carbonyl atom as the SOM. The predicted SOM is marked in orange, while the true SOM is indicated in blue. Future versions will correct this error.

or closure, and molecular rearrangement. These processes complicate the accurate AAM between substrate and metabolite, often resulting in incorrect annotations. In Figure S9, the true SOMs are indicated in blue, while the predicted SOMs include both the correct (blue) and additional incorrect (orange) atoms. In Figure S10, the true SOMs are shown in blue, whereas the incorrectly predicted SOMs are marked in orange.

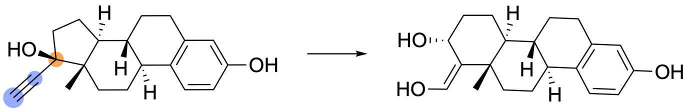

Figure S9: Example of an incorrectly annotated "complex" reaction involving an oxidation reaction followed by a subsequent molecular rearrangement. The true sites of metabolism (SOMs) are highlighted in blue, while the predicted SOMs include both the correct atoms (blue) and additional incorrect atoms (orange).

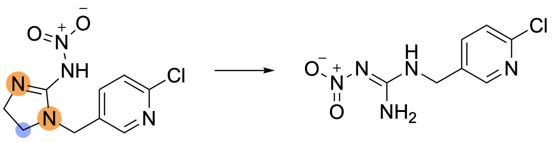

Figure S10: Example of a misannotation in a "complex" reaction involving a ring opening. The true SOMs are shown in blue, whereas the predicted SOMs are shown in orange.

S5 Evaluating AutoSOM's Potential for Leveraging Dormant Metabolic Data: aweSOM's and FAME3R's Hyperparameters

In a series of experiments detailed in the main body of this work in Section 4.4, we evaluated whether AutoSOM's automatically generated SOM labels are accurate enough to train models that match the performance of those trained on expert-labeled data. To this end, we retrained two state-of-the-art SOM prediction models—FAME3R[4] and aweSOM[5]—on two versions of the MetaQSAR training set: one labeled by experts, and the other labeled by AutoSOM.

Below, we detail the hyperparameters used for each model during training:

- FAME3R
  - Framework: CDPKit[6] (descriptors) and scikit-learn[7] (classifier)
  - Algorithm: random forest
  - Number of trees: 100
  - Splitting criterion: Gini impurity
  - Maximum depth of the tree: None
  - Minimum number of samples required to split an internal node: 2
  - Minimum number of samples required per leaf: 1
  - Feature selection: Square root of the number of features
  - Bootstrap: true
  - Class balancing: enabled (using balanced sample weighting)

- awESOM
  - Framework: PyTorch Geometric
  - Architecture: awESOM is an ensemble of 10 Graph Neural Network (GNN)-models, each trained with different random initializations. Each model consists of three core modules: a convolutional module, a molecular context pooling module, and a classification module. The convolutional module includes four GINE layers[8] of size 500, interspersed with batch normalization and LeakyReLU activation. The molecular context-pooling module generates a graph-level representation via additive pooling. This pooled representation is concatenated to all node-level representations in the graph, effectively doubling their dimensionality. The classification module consists of two fully-connected layers with 888 neurons, interspersed by batch normalization, LeakyReLU[9] activation, and dropout layers with dropout probability 0.2, followed by a final fully connected layer that maps the latent representation to a probabilistic output using the sigmoid function.
  - Optimizer: AdamW[10]
  - Initial learning rate: 3.96×10^{-5}
  - Weight decay: 1.25×10^{-5}
  - Batch size: 32
  - Loss function: binary cross-entropy with positive class weight 2.11
  - Number of epochs: 40

All hyperparameters were selected based on prior benchmarking and follow the configurations used in earlier studies for FAME3R[11] and awESOM,[5] with no additional tuning on the experimental setup used in this study.

References

(1) Pedretti, A.; Mazzolari, A.; Vistoli, G.; Testa, B. MetaQSAR Database. Download: 2023-10-01.

(2) Litsa, E. E.; Das, P.; Kavraki, L. E. Prediction of Drug Metabolites Using Neural Machine Translation. *Chem. Sci.* **2020**, *11*, 12777–12788.

(3) Litsa, E. E.; Das, P.; Kavraki, L. E. Metabolite Translator (MetaTrans). `https://github.com/KavrakiLab/MetaTrans`, 2020; Accessed: 2025-03-10.

(4) Chen, Y.; Seidel, T.; Jacob, R. A.; Hirte, S.; Mazzolari, A.; Pedretti, A.; Vistoli, G.; Langer, T.; Miljković, F.; Kirchmair, J. Active Learning Approach for Guiding Site-of-Metabolism Measurement and Annotation. *J. Chem. Inf. Model.* **2024**, *64*, 348–358.

(5) Jacob, R. A.; Wieder, O.; Chen, Y.; Mazzolari, A.; Bergner, A.; Schleifer, K.-J.; Kirchmair, J. AweSOM: a GNN-based Site-of-Metabolism Predictor with Aleatoric and Epistemic Uncertainty Estimation. *ChemRxiv* **2025**, 10.26434/chemrxiv–2024–pzmqt–v2.

(6) Seidel, T. Chemical Data Processing Toolkit Source Code Repository. `https://github.com/molinfo-vienna/CDPKit`, Accessed: 2025-03-10.

(7) Pedregosa, F. et al. Scikit-learn: Machine Learning in Python. *JMLR* **2011**, *12*, 2825–2830.

(8) Hu, W.; Liu, B.; Gomes, J.; Zitnik, M.; Liang, P.; Pande, V.; Leskovec, J. Strategies for Pre-training Graph Neural Networks. *Proceedings of the 8th International Conference on Learning Representations, Addis Ababa, Ethiopia, April 26-30* **2020**, 10.48550/arXiv.1905.12265.

(9) Maas, A. L.; Hannun, A. Y.; Ng, A. Y. Rectifier Nonlinearities Improve Neural Network

Acoustic Models. *Proceedings of the 30th International Conference on Machine Learning, Atlanta, United States of America, June 16-21* **2013**,

(10) Loshchilov, I.; Hutter, F. Decoupled Weight Decay Regularization. *Proceedings of The 7th International Conference on Learning Representations, New Orleans, United States of America, May 6-9* **2019**, 10.48550/arXiv.1711.05101.

(11) Šícho, M.; Stork, C.; Mazzolari, A.; de Bruyn Kops, C.; Pedretti, A.; Testa, B.; Vistoli, G.; Svozil, D.; Kirchmair, J. FAME 3: Predicting the Sites of Metabolism in Synthetic Compounds and Natural Products for Phase 1 and Phase 2 Metabolic Enzymes. *J. Chem. Inf. Model.* **2019**, *59*, 3400–3412.
